# Comparative Phosphoproteomic Analysis Reveals the Response of Starch Metabolism to High-Temperature Stress in Rice Endosperm

**DOI:** 10.3390/ijms221910546

**Published:** 2021-09-29

**Authors:** Yuehan Pang, Yaqi Hu, Jinsong Bao

**Affiliations:** Institute of Nuclear Agricultural Sciences, College of Agriculture and Biotechnology, Zhejiang University, Zijingang Campus, Hangzhou 310058, China; pyh_tt@163.com (Y.P.); 11916013@zju.edu.cn (Y.H.)

**Keywords:** rice endosperm, phosphorylation, high temperature, sucrose and starch metabolism, starch biosynthesis

## Abstract

High-temperature stress severely affects rice grain quality. While extensive research has been conducted at the physiological, transcriptional, and protein levels, it is still unknown how protein phosphorylation regulates seed development in high-temperature environments. Here, we explore the impact of high-temperature stress on the phosphoproteome of developing grains from two indica rice varieties, 9311 and Guangluai4 (GLA4), with different starch qualities. A total of 9994 phosphosites from 3216 phosphoproteins were identified in all endosperm samples. We identified several consensus phosphorylation motifs ([sP], [LxRxxs], [Rxxs], [tP]) induced by high-temperature treatment and revealed a core set of protein kinases, splicing factors, and regulatory factors in response to high-temperature stress, especially those involved in starch metabolism. A detailed phosphorylation scenario in the regulation of starch biosynthesis (AGPase, GBSSI, SSIIa, SSIIIa, BEI, BEIIb, ISA1, PUL, PHO1, PTST) in rice endosperm was proposed. Furthermore, the dynamic changes in phosphorylated enzymes related to starch synthesis (SSIIIa-Ser94, BEI-Ser562, BEI-Ser620, BEI-Ser821, BEIIb-Ser685, BEIIb-Ser715) were confirmed by Western blot analysis, which revealed that phosphorylation might play specific roles in amylopectin biosynthesis in response to high-temperature stress. The link between phosphorylation-mediated regulation and starch metabolism will provide new insights into the mechanism underlying grain quality development in response to high-temperature stress.

## 1. Introduction

Rice is an agriculturally major cereal crop worldwide. The yield and quality of rice are often severely affected by heat stress, and this phenomenon is further aggravated with the intensified global warming [1]. During endosperm development, exposure to high-temperature environment results in an accelerated filling rate of rice grains, which eventually leads to poor grain quality and a severe reduction in yield [2,3]. The impact of high-temperature stress on physiological metabolism has been intensively investigated [2,4]. Furthermore, transcriptome and proteome profiles in rice endosperm have been used to explore differences under high-temperature stress at gene [5,6] and protein [7,8,9] expression levels. However, a notable paucity exists focusing specifically on phosphorylation-mediated regulation under heat stress during the grain-filling stage.

It is now well accepted that phosphorylation plays a vital role in the regulation of many intracellular processes during plant growth and development. The possible role of protein phosphorylation in the formation of a protein complex participating in starch synthesis in wheat endosperm was first proposed by Tetlow et al. [10,11]. Later studies conducted in maize have also revealed a vital regulation role of phosphorylation in forming a starch-synthesizing protein complex [12,13]. Further examinations of individual enzymes basically came from maize endosperm [13,14]. In particular, Makhmoudova et al. identified the phosphorylation status of SBEIIb at three sites (Ser 286, Ser297, and Ser649) by Ca^2+^-dependent protein kinase [15]. With the development of mass-spectrometry-based techniques [16], several sites of phosphorylation that may regulate starch biosynthesis were discovered in cereal endosperm [17,18], despite the real functions of those phosphosites being still unclear [19]. Qiu et al. identified some phosphorylated proteins in rice pistils and seeds and focused on the differentially phosphorylated proteins at early seed development [20]. Developing rice seeds undergo active cell division and differentiation at the early stage, and 6 to 20 days after flowering (DAF) is the critical period for grain filling and starch accumulation [21]. Hence, there has been little systematic discussion at the phosphorylation level from rice endosperm as compared to the other main crops, especially at the critical periods of starch accumulation. Although several phosphosites involved in starch synthesis were identified in our previous research [22], an in-depth investigation under abiotic stress is necessary to gain a more comprehensive understanding of the regulation pathway.

Here, a label-free quantitative phosphoproteomic analysis was applied to examine the heat-induced phosphorylation change in two rice varieties with different starch qualities. As a result, a series of phosphorylation motifs, enzymes, protein kinases, splicing factors, and other potential regulators involved in seed development were revealed, particularly the phosphoproteins involved in starch metabolism. The properties of phosphorylation sites involved in starch synthesis and their change trends in response to heat stress were comprehensively explored through sequence alignment and site conservation analysis. Western blot with site-specific phosphopeptide antibodies was used to verify and explore the dynamic change in phosphorylation related to starch synthesis under high-temperature stress. Taken together, an in-depth understanding of phosphorylation-mediated regulation in rice endosperm under heat stress will shed new light on thermal signaling transduction and functional phosphosites related to starch metabolism.

## 2. Results

**Figure 1:**
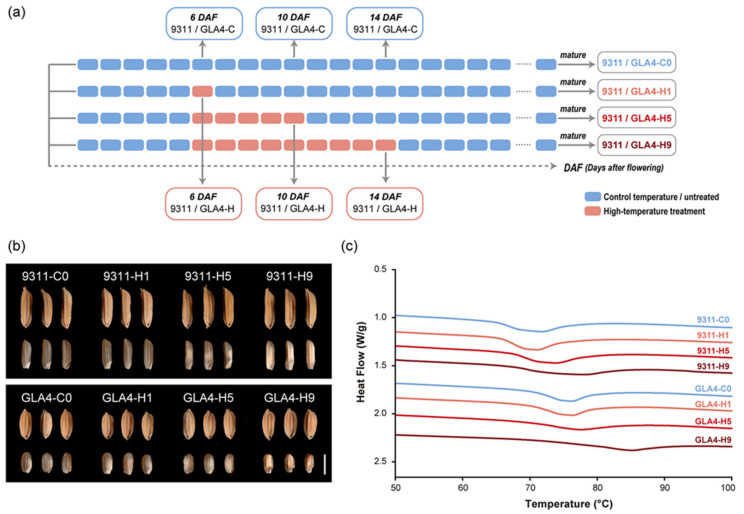
Effect of high-temperature stress on the rice grain. (**a**) Schematic diagram of the experimental design and sampling time points during the grain-filling period with high-temperature treatment. (**b**) Effect of three high-temperature treatments on grain morphologies of 9311 and GLA4. Scale bar = 5 mm. (**c**) Impact of three high-temperature treatments on DSC thermograms of 9311 and GLA4.

We sought to assess dynamic changes in protein phosphorylation in the developing rice seed under high-temperature stress. The control and high-temperature treatment groups were designed for two indica rice varieties, 9311 and Guangluai4 (GLA4). In the treatment group, heat stress was performed on the fifth DAF, which prevented the potential effects of high temperature on pollination and seed setting at the early developmental stage. In addition, the 6, 10, and 14 DAF were designed as sampling time points, corresponding to the 1, 5, and 9 days of high-temperature treatment, respectively (Figure 1a and Figure S1). The average temperature of the treatment group (HT; 30–38 °C; Figure S1) was 10 °C higher than that of the control group (CT; 20–28 °C; Figure S1), while other parameters were kept the same between two artificial climatic chambers. Immature endosperm samples were analyzed by LC-MS/MS and Western blot, and the corresponding mature seeds were harvested for the determination of starch thermal properties.

### 2.1. Dynamic Changes in Rice Grain Appearance and Thermal Properties under High-Temperature Treatment

Grain chalkiness is an indicator of abnormally developed endosperm and the most sensitive trait in response to heat stress [2,3]. It is evident that the chalkiness degree gradually increased with increasing days of high-temperature treatment (Figure 1b). After 5 days of treatment, noticeable chalkiness changes were observed in both varieties, demonstrating the effectiveness of high-temperature treatment (Figure 1b). Besides, shrunken grains were found after 9 days of treatment, especially in the GLA4-H9 group (Figure 1b).

The thermal properties of the flour samples were determined by differential scanning calorimetry (DSC) (Figure 1c and Table S1). All GLA4 samples had significantly higher gelatinization temperatures than the 9311 samples (Figure 1c), indicating a dramatic difference between the two varieties in starch physicochemical properties, which is in good agreement with our previous results [22]. One-day treatment only appeared to affect the thermal properties of 9311 plants (Table S1). After 5 days of exposure to high-temperature stress, a significantly higher gelatinization temperature was observed in both varieties, and this phenomenon was more noticeable after 9 days of treatment (Table S1). 

Overall, results of grain chalkiness and thermal properties indicated that high-temperature treatment for 5 days is sufficient to cause irreversible damage to rice grain quality. Accordingly, samples collected at 10 DAF with 5 days of treatment were the optimal treated group for subsequent phosphoproteomic analysis.

### 2.2. Phosphoproteins Identified in Rice Endosperm

To elucidate how heat stress influences rice endosperm at the phosphorylation level, a label-free analysis was performed to quantify phosphoproteome from two indica rice cultivars under normal and high-temperature conditions. As depicted in Figure S2a, samples of 9311-C, 9311-H, GLA4-C, and GLA4-H were collected, pretreated, lysed, digested, and enriched, and then analyzed by LC-MS/MS. To validate the accuracy of MS data, we confirmed the mass error of all identified peptides and found the distribution satisfied an expected error control (Figure S2b). Meanwhile, the distribution of peptide length was checked to ensure that sample preparation reached standard conditions (Figure S2c).

**Figure 2:**
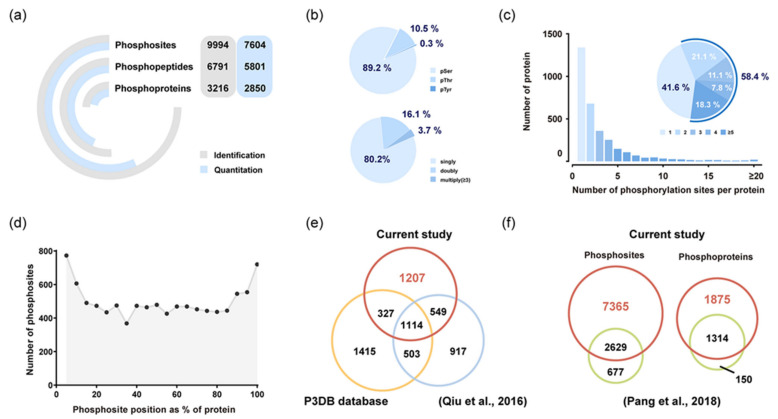
Characteristics of the phosphoproteome of rice endosperm at the critical grain-filling stage. (**a**) Summary of phosphoproteome analysis in rice endosperm. (**b**) Distribution of the number of phosphates and phospho-amino acid residues for all quantifiable phosphopeptides. (**c**) Frequency distribution of phosphoproteins according to the number of phosphosites identified. (**d**) Positional distribution of the identified phosphosites in protein sequences. (**e**) Overlap of the identified phosphoproteins in our study with phosphoproteins in the japonica datasets—the P3DB database [24] and the previously published phosphoproteome of japonica rice endosperm [20]. (**f**) Comparative analysis of phosphosites and phosphoproteins between the current phosphoproteome and our previous research [22].

In all endosperm samples, our workflow led to the identification of 9994 phosphosites located on 3216 proteins, of which 7604 phosphosites were quantifiable (Figure 2a and Table S2). The majority of the phosphosites quantified were identified as serine (89.2%), followed by threonine (10.5%) and tyrosine (0.3%) residues (Figure 2b). Among the 5801 peptides quantified, singly phosphorylated peptides were dominant (80.2%), and around 3.7% peptides carried three or more phosphorylation modifications (Figure 2b). The number of phosphorylated sites in a single protein also varied considerably, with the range from 1 to 55 residues (Figure 2c). Over half phosphoproteins possessed two or more sites, indicating the functional importance of proteins with multiple phosphosites in regulatory networks. Distribution of the phosphorylation sites in specific protein regions suggested that N- and C-terminal regions are preferentially phosphorylated (Figure 2d), which were consistent with data obtained in Arabidopsis anthers [23]. A comparative analysis between our dataset and the japonica dataset (P3DB database) [24] and the previously published phosphoproteome of japonica rice endosperm [20] was performed (Figure 2e). Over 1000 phosphorylated proteins were common to all three datasets, and 1207 phosphoproteins were newly identified in this study. Moreover, we discovered 7365 novel phosphosites involved in 1875 phosphoproteins compared with our previous research (Figure 2f) [22].

### 2.3. A Temperature-Regulated Rice Endosperm Phosphoproteome

**Figure 3:**
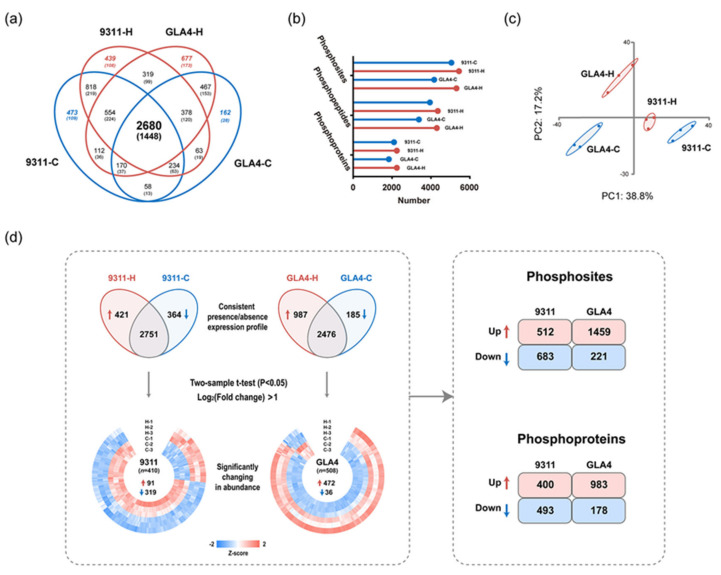
A temperature regulated rice endosperm phosphoproteome. (**a**) Venn diagram depicting the comparison of phosphosites (phosphoproteins) from four sample groups. (**b**) Number of phosphosites, phosphopeptides, and phosphoproteins detected in 9311-C, 9311-H, GLA4-C, and GLA4-H. (**c**) Principal component analysis (PCA) based on phosphorylation intensity across all four sample groups with three biological repetitions. (**d**) Differentially expression profiles of phosphosites (phosphoproteins) in 9311 and GLA4 under high-temperature stress. The expression profiles of selected phosphosites (*p* < 0.05, log_2_ (fold change) >1) were normalized using the Z-score and presented in a heatmap. In each variety, phosphosites (phosphoproteins) with a consistent presence/absence expression pattern and significantly regulated from the statistical test were combined for subsequent comparative analysis.

To detect possible changes in the phosphoproteome attributable to heat stress, we then performed label-free quantification analysis on all quantifiable phosphosites within our dataset. Only 2680 common phosphosites were quantifiable for all sample groups due to reversible phosphorylation induced by high temperature (Figure 3a). More phosphosites, phosphopeptides, and phosphoproteins were identified in 9311-H and GLA4-H groups (Figure 3b), suggesting that exposure to heat stress may increase the phosphorylation events in rice endosperm. Principal component analysis (PCA) showed that three repeats of each sample clustered together, and four groups were clearly separated (Figure 3c). Pearson’s correlation coefficients were also generated, suggesting good reproducibility and consistency between replicates (Figure S2d).

To detect possible changes in the phosphoproteome attributable to heat stress, we then performed label-free quantification (LFQ) analysis on all phosphosites identified within our dataset (Figure 3d). Where LFQ values were missing, the data were filtered to identify those phosphosites with a consistent presence/absence expression pattern. These analyses yielded 421 phosphosites that were only present in 9311-H and 364 that were only present in 9311-C (Figure 3d and Table S3). Similarly, 987 differentially abundant phosphosites were present in GLA4-H and 185 phosphosites that only occurred in GLA4-C (Figure 3d and Table S4). Beyond that, a total of 410 and 508 significantly changed phosphorylation sites (*p* < 0.05, log_2_ (fold-change) >1) were screened out in 9311 and GLA4, respectively, where LFQ data was available in both conditions (Figure 3d and Tables S5 and S6). For subsequent comparative analysis, phosphorylation sites that were uniquely identified in either condition and significantly regulated from the statistical test were combined and divided into four groups (9311-Up, 9311-Down, GLA4-Up, and GLA4-Down; Figure 3d). The number of significantly down-regulated phosphosites was far greater than up-regulated phosphosites in the 9311 variety. However, the opposite trend was observed in GLA4 plants. Comparing four sets of differential phosphorylated sites, we found only 132 phosphosites were commonly up-regulated in both rice varieties and 24 were commonly down-regulated (Figure S3a). In addition, there were 74 phosphosites showing completely opposite regulatory trends in two cultivars induced by high temperature (Figure S3a). When all significantly changed phosphosites corresponded to the specific protein, comparison results become even more complicated. There was a compelling phenomenon that 39 phosphoproteins of 9311 and 43 of GLA4 displayed a combination of up- and down-regulated phosphosites (Figure S3b). It is possible, therefore, that the status of these phosphosites was directly controlled by associated kinases and phosphatases.

### 2.4. Regulation of Phosphorylation Motifs and Kinases

**Figure 4:**
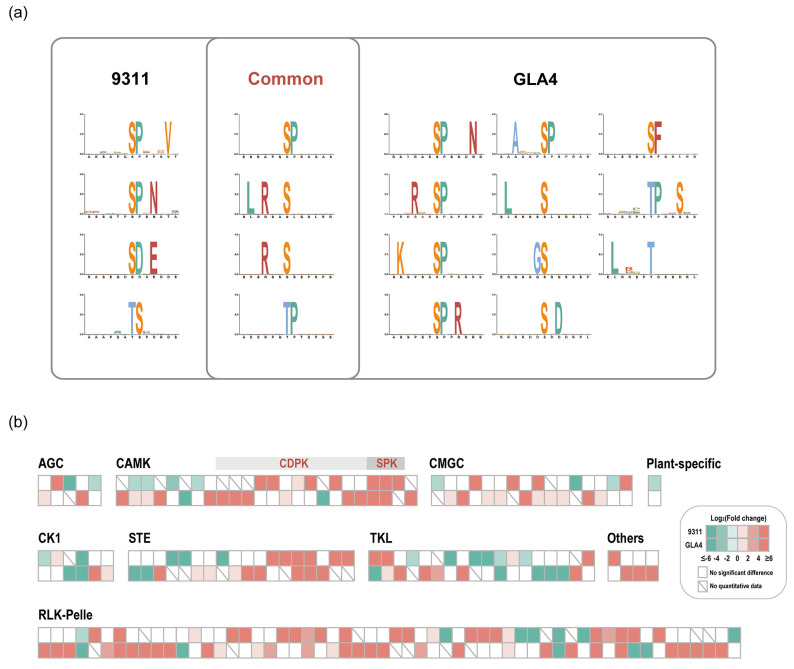
Motif enrichment analysis of differential phosphopeptides in 9311 and GLA4 (**a**) and phosphorylated kinases with significant differences in rice endosperm under high-temperature stress (**b**).

The in vivo phosphorylation status induced by heat stress is often inseparable from protein kinase activity, which is usually regulated by upstream kinases or autophosphorylation. Up to now, few studies have examined the association between high-temperature response and protein phosphorylation involved in the signaling pathway [25,26]. It is now well established from a variety of studies that candidate substrates for the specific kinase are identified based on motif analysis [27]. A detailed investigation with a focus on potential phosphorylation motifs was, therefore, first conducted. We retrieved 8 and 15 over-represented motifs from the Ser-/Thr-containing differential phosphopeptides in 9311 and GLA4, respectively (Figure 4a). 

Both 9311 and GLA4 shared a number of consensus motifs ([sP], [LxRxxs], [Rxxs], [tP]; Figure 4a). Motifs presented are the results induced by multiple kinases, which were activated by high-temperature stress. Proline-directed motifs, such as [sP] and [tP], were recognized by kinases CDK, RLK, RLCK, MPK, SnRK2, CDPK, and SLK [27]. Note that [RxxS], which could be recognized by CDPKs and SnRKs, was also the 14-3-3 binding motif [28,29]. [LxRxxs] was known to be targeted by CDPKs [29]. The acidic motif [SDxE] was unique to 9311 and known to be targeted by CDPK, RLK, and AGC [27]. Notably, it is plausible that CDPKs were the key kinases in response to high-temperature stress. because all consensus motifs and [SDxE] were potential substrates for CDPKs. Apart from common motifs, the phosphosites from GLA4 samples yielded more enriched motifs than 9311, indicating a more complicated kinase system in response to heat stress in GLA4 plants (Figure 4a). In support of this, we found the number of up-regulated phosphosites in GLA4 kinases is considerably greater than that in 9311 kinases (Figure 3d).

In total, 192 protein kinases with 691 phosphorylated sites were identified in our phosphoproteomic dataset (Table S7), including RLKs (87, 45.3%), TKL (22, 11.5%), CMGC (21, 10.9%), and CAMK (20, 10.4%) [30]. Further enrichment analysis indicated that the family of TKL, CMGC, CAMK, STE, and CK1 was over-represented (Figure S4a). Under heat stress, only 47 phosphosites of kinases showed significant up-regulation in 9311, whereas the up-regulated sites in GLA4 were up to 77 (Table S7). Of the 148 phosphosites that were significantly regulated (Figure 4b), only 16 phosphosites showed the same regulatory trend in both varieties, while 4 phosphosites showed opposite regulatory trends (Figure S4b). In this sense, it could reasonably explain the vast difference in the phosphorylation regulation pattern between the two varieties (Figure 3d).

### 2.5. Functions for Differentially Phosphorylated (DP) Proteins

GO enrichment was applied to analyze the DP phosphoproteins to obtain an overview of the phosphorylation events during grain development. As expected, the up-regulated phosphoproteins in both 9311 and GLA4 were highly enriched in terms of heat response, such as heat acclimation, response to heat [26], and response to temperature stimulus. Among proteins involved in the heat response, phosphorylation levels of 12 heat shock proteins (HSPs) increased significantly under heat stresses. Of these, five phosphorylated sites were found common to both varieties (Tables S3–S7). 

In the biological process, the most interesting aspect was the metabolic process in which abundant phosphoproteins in GLA4-Up and 9311-Down were enriched (Figure S5a and Table S8). Besides, the up-regulated functional phosphoproteins of 9311 were enriched in glucan polysaccharide and transduction phosphorylation, while the down-regulated ones were over-represented in negative regulation. From the molecular function perspective, ATPase activity, kinase activity, phosphotransferase activity, and binding for GTP and nucleic acid binding were mainly enriched, indicating the importance of kinases, phosphatases, and transcription factors in the phosphorylation regulatory network (Figure S5b and Table S9). Figure S6 provides the summary statistics for the phosphorylated TFs. Overall, 140 phosphorylated TFs that were divided into 35 families were identified [31]. The largest fraction was identified as the C3H family (21, 15%), followed by bZIP (18, 12.9%) and Trihelix (11, 7.9%) (Figure S6). Meanwhile, these phosphorylated TF families were highly enriched when using the total TFs identified in the indica rice database as a reference. 

KEGG database annotation was then applied to predict the potential metabolic pathways. In the 9311 group, DP phosphoproteins were mainly over-represented in the pathway of spliceosome, transcription, starch and sucrose metabolism, and aminoacyl-tRNA biosynthesis (Figure S5c). In the GLA4 group, three genetic information pathways (ribosome biogenesis in eukaryotes, mismatch repair, and nucleotide excision repair) and the spliceosome pathway were enriched (Figure S5c). 

### 2.6. Phosphoproteins Identified in Starch Metabolism

**Figure 5:**
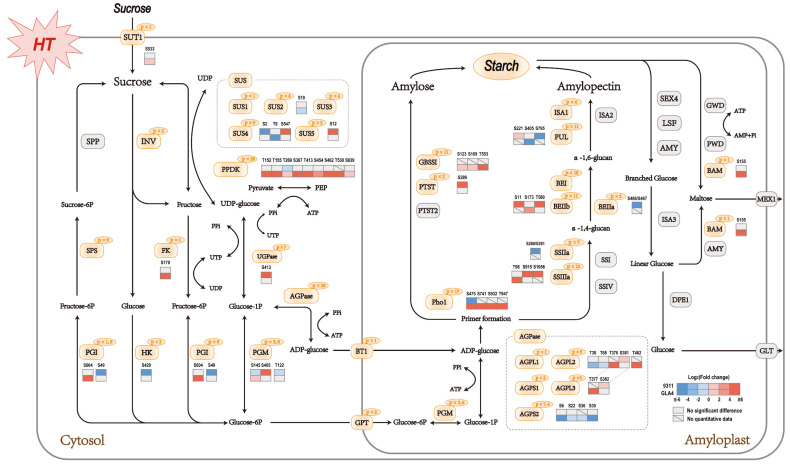
Sucrose and starch pathway at the phosphorylation levels in rice endosperm under high-temperature stress. Orange and gray shadings represent phosphorylated and non-phosphorylated proteins, respectively. The number of phosphosites is shown at the top right of rounded boxes. Sucrose hydrolysis: SUS, sucrose synthase; INV, invertase; SPP, sucrose-phosphate phosphatase; SPS, sucrose-phosphate synthase; FK, fructokinase; HK, hexokinase; PGI, glucose-6-phosphate isomerase; PGM, phosphoglucomutase; UGPase, UDP-glucose pyrophosphorylase. Starch synthesis: AGPase, ADP-glucose pyrophosphorylase; GBSSI, granule-bound starch synthase I; PTST, protein targeting to starch; SSI, SSIIa, SSIIIa, and SSIV, starch synthase I, IIa, IIIa, and IV, respectively; BEI, BEIIa, and BEIIb, starch branching enzyme I, IIa, and IIb, respectively; ISA1, ISA2, isoamylase isoform 1 and 2, respectively; PUL, pullulanase; Pho1, plastidial phosphorylase. Starch hydrolysis: AMY, α-amylase; BAM, β-amylase; LSF, like sex four; SEX4, starch excess 4; ISA3, isoamylase isoform 3; GWD, glucan water dikinase; PWD, phosphoglucan water dikinase; DPE1, disproportionating enzyme 1. Protein transport: SUT1, sucrose transporter; BT1, small-solute transporter; GPT, phosphometabolite transporter; MEX1, maltose transporter; GLT, glucose transporter.

There is no doubt that sucrose and starch metabolism was the most noteworthy pathway with a large number of phosphoproteins involved (Table S10). A systematic and detailed investigation was then conducted of the specific proteins involved in sucrose and starch metabolism (Figure 5). The critical functions of phosphoproteins involved can be listed as follows: sucrose hydrolysis (SUS, INV, SPS, FK, HK, PGI, PGM, and UGPase), starch synthesis (AGPase, GBSSI, SSIIa, SSIIIa, BEI, BEIIb, ISA1, PUL, Pho1, and PTST), starch hydrolysis (BAM), and protein transport (SUT1, BT1, and GPT). To assess how high-temperature stress affects the crucial pathway, the significantly differential phosphosites in 9311 and GLA4 were displayed in the heatmap of specific proteins (Figure 5). From the perspective of sucrose hydrolysis, almost all enzymes that provide G1P for starch synthesis possessed phosphorylation sites (Figure 5). Reversible phosphorylation events were flexibly regulated by high-temperature stress in starch biosynthesis, whereas only one enzyme possessed phosphorylation modification in the starch degradation pathway (Figure 5).

In starch biosynthesis, the key rate-limiting enzyme AGPase, including AGPL1, AGPL2, AGPL3, AGPS1, and AGPS2, was phosphorylated among all sample groups (Figure 5 and Table S10). Interestingly, heat stress down-regulates the phosphosites of AGPL2 located in the N-terminal and up-regulates the sites in C-terminal, even though those sites in 9311 and GLA4 were found at different positions (Figure 5). In GLA4, most phosphosites of AGPS2 were down-regulated, probably owing to the regulation of protein abundance of AGPase under high-temperature stress [7].

**Figure 6:**
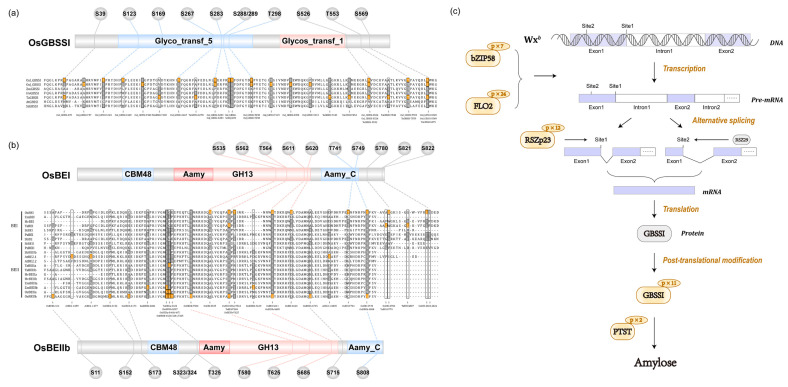
Domain structure and amino acid sequence alignments of GBSSI (**a**) and BEs (**b**). Residues in yellow indicate the phosphorylation site. Non-phosphorylated residues are shown in dark gray. (**c**) Potential effect of phosphorylation regulation on amylose biosynthesis by regulatory factors (OsbZIP58 and FLO2), RSZp23, GBSSI, and PTST.

Our study identified a large number of phosphorylation sites in GBSSI and found that 6 phosphosites were located at the glycosyltransferase 5 domain (Figure 6a). However, no valid phosphorylation intensity value was detected in 9311 due to a relatively lower phosphorylation level caused by the low abundance of GBSSI protein (Figure S7 and S8). In GLA4 groups, it is obvious that exposure to heat stress resulted in increasing GBSSI phosphorylation intensity at S123, S169, and S553. Phosphorylation events were prevalent in SSIIa and SSIIIa with more than 20 phosphosites but absent in SSI as well as SSIV (Figure 5 and Figure S9). In GLA4 groups, heat stress triggered the increasing phosphorylation intensity of SSIIIa-T98. Likewise, the phosphorylation intensity of S915 and S1058 was greatly enhanced in the 9311 heat-stressed group. SSIIIa-S915 was conserved among all plants’ SSIII and located at the CBM53 domain (Figure S9), which has been shown to be necessary for enzyme activity and affinities toward various glucans. Interestingly, a single peptide of SSIIa could only be detected under normal conditions in 9311, although the phosphosite could not be accurately localized (either S260 or S261; Figure S9).

Three BE isozymes possessed the same CBM48, GH13, and Aamy_C domains (Figure 6b). However, the phosphorylation events among BEs were somewhat discordant, with 10, 2, and 11 phosphosites involved in BEI, BEIIa, and BEIIb (Figure 6b and Table S10), respectively. There was an intriguing correlation among OsBEI, OsBEIIa, and OsBEIIb in that five serine residues of the three isozymes were phosphorylated at the same position (Figure 6b). More concretely, the S562, S611, and S749 of OsBEI corresponded to the S562, S685, and S808 of OsBEIIb, respectively, and the OsBEIIb-S323/324 corresponded to OsBEIIa-S466/S467 (Figure 6b). In the 9311 group, heat stress triggered phosphorylation at S11 and T580 of BEIIb and exerted suppressive effects on BEIIa-S467 (Figure 5). Only one phosphosite (BEIIb-S173) in GLA4 was up-regulated when exposed to heat stress (Figure 5).

Among 11 phosphosites identified in PUL, phosphoserine 221 in 9311, within the PULN2 domain of PUL, was 4.9-fold up-regulated at high temperature. Another phenomenon we observed is that S795 and S405 appeared only in 9311-C and GLA4-C, respectively (Figure 5). We found a widespread occurrence of Pho1 phosphorylation events in rice endosperm, and three residues were phosphorylated in the rice L80 region (Figure S10 and Table S10). Here, phosphoserine 376 in 9311 disappeared as the temperature increased (Figure 5). Curiously, this phosphosite in GLA4 could only be detected under high-temperature conditions. Besides, another three phosphosites (S741, S932, T947) were significantly up-regulated in GLA4 (Figure 5).

Apart from the enzymes mentioned above, phosphorylated regulatory factors related to starch metabolism are given in Figure S11a. Among all seven phosphosites of OsbZIP58, three sites (S46, S183, S277) of GLA4 samples were up-regulated, while no significantly regulated phosphosites were observed in 9311. There were 24 residues phosphorylated at FLO2. Specifically, 9311 witnessed different degrees of significant down-regulation in nine phosphosites, while for GLA4, only one phosphosite increased considerably. Phosphoserine 277 is located within the bZIP_1 domain, whose function is to mediate sequence-specific DNA binding properties and the leucine zipper. 

### 2.7. Dynamic Change in Phosphorylation Status Related to Starch Synthesis 

**Figure 7:**
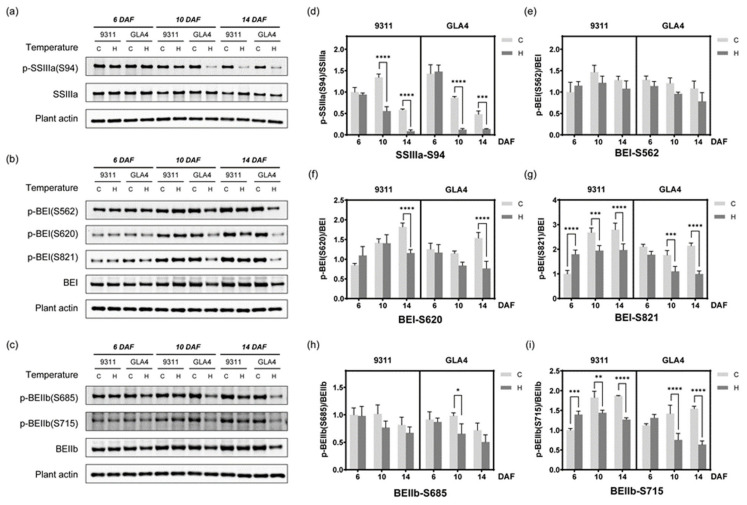
Western blot assay of some phosphorylated proteins. (**a**–**c**) Western blot assay of the phosphorylation of SSIIIa-S94, BEI-S562, BEI-S620, BEI-S821, BEIIb-S685, and BEIIb-S715. The best blot of three independent experiments is shown here. The results of the independent experiments followed a similar trend in expression. Uncropped gels are shown in Figure S19. (**d**–**i**) Evaluation of the relative phosphorylation intensity of starch-synthesis-related enzymes at the three grain-filling stages under high-temperature stress. * *p* < 0.05, ** *p* < 0.01, *** *p* < 0.001, and **** *p* < 0.0001.

To further validate and explore the dynamic change in the phosphorylation status involved in starch synthesis under high-temperature stress, site-specific phosphopeptide antibodies (BEI-Ser562, BEI-Ser620, BEI-Ser821, BEIIb-Ser685, BEIIb-Ser715, SSIIIa-Ser94) were prepared, which were identified in a previous study [22] and the present study (Table S11). The mass spectrum of synthetic peptides ensures the quality of blocking peptides (Figure S12). Only when specific phosphopeptides were incubated with antibodies did the specific band disappear (Figure S13), confirming the high efficiency and specificity of the phosphor-antibodies. The phosphorylation status of SSIIIa (SSIIIa-S94), BEI (BEI-Ser562, BEI-Ser620, BEI-Ser821), and BEIIb (BEIIb-Ser685, BEIIb-Ser715) was further confirmed by Western blot analysis (Figure 7a–i).

Starch synthase IIIa (SSIIIa) has the second-highest starch synthase (SS) activity and plays a critical role in forming long B chains, most notably B2 and B3 chains [32,33]. Considering the phosphorylation event of SSIIIa-S94 was identified multiple times in rice endosperm [20,22], we speculated that S94 is a crucial site for protein function and prepared a site-specific phosphopeptide antibody for Western blot (Table S11). To eliminate the impact of protein abundance on the phosphorylation level, the relative phosphorylation intensity was used to evaluate the regulation of the modification level of the phosphosite. As shown in Figure 7d, the relative phosphorylation intensity of SSIIIa-S94 decreased with the growth period, and high-temperature treatment exacerbated the inhibitory tendency. In the meantime, SSIIIa protein expression witnessed significantly and relatively mild rises in 9311 and GLA4 at 10 DAF, respectively (Figure 7a and Figure S8), which was inversely associated with the phosphorylation status of S94. Results from immunoblotting analysis at 10 DAF were consistent with the trend tested by the mass spectrum except for the GLA4-C group (Figure S14). We attributed this slight inconsistency to the different characteristics of the normalization methods. In other words, high temperature severely inhibits the phosphorylation level of SSIIIa-S94, and the increase in protein expression does not appear to compensate for the phosphorylation reduction. 

Branching enzymes (BEs) including BEI, BEIIa, and BEIIb, are fundamental to form a distinct fine structure of amylopectin [34]. Given the repeated identification in the phosphoproteome [22] and high probabilities of these phosphosites, it is worth to determine the phosphorylation intensity of BEI-S562, BEI-S620, BEI-S821, BEIIb-S685, and BEIIb-S715 (Figure 7b,c). Large proportion of missing values and poor repeatability of LC/MS-MS data in these specific phosphosites made the trend difficult to evaluate (Figure S14), and statistics from immunoblotting analysis may compensate for the deficiency. A significant decline in both varieties was observed in the relative phosphorylation intensity of BEI-S620, BEI-S821, and BEIIb-S715 at 14 DAF (Figure 7f,g,i). Besides, evident alterations in the phosphorylation status of BEI-S821 and BEIIb-S715 already appeared at 6 DAF (Figure 7g,i). On the contrary, BEI-S562 and BEIIb-S685 changed their phosphorylation status based on the BE protein expression with temperature stimulus (Figure 7e,h).

To gain a better understanding of the phosphorylation-mediated regulation mode of starch-synthesis-related enzymes under abiotic stress, we counted the number of significant differences for each of the phosphosites mentioned above and analyzed their relationship with site conservation (Table S12). A meaningful outcome may be that phosphorylation levels of conserved phosphosites (BEI-S562 and BEIIb-S685) were not significantly affected by heat stress, while significant phosphorylation changes were observed in non-conserved phosphosites (SSIIIa-S94, BEI-S620, BEI-S821, and BEIIb-S715) at different periods of grain development (Table S12).

## 3. Discussion

Generally, japonica rice is used as the primary material for phosphoproteomic investigations [20,24]. However, research focus on indica rice seems even more necessary because it is the most widely cultivated rice in Asia, with over 70% of rice production worldwide. As shown in Figure 2e, 1114 phosphoproteins are common to all three datasets, whose phosphorylation status is ubiquitous across different rice varieties or tissues (Figure 2e). Notably, 1207 phosphoproteins were newly identified in this study, substantially filling the missing information in the rice phosphorylation database. In particular, a large number of phosphoproteins related to starch metabolism, including abundant enzymes, transcription factors, and kinases, were newly identified. Compared with our previous research [22], the current phosphoproteome increased 73.7% identification (7365 phosphosites; Figure 2f), which likely benefited from the improved experimental technique and stress treatments. The current research covered 89.8% phosphoproteins detected previously (Figure 2f), further confirming the reliability of the experiment. Taken together, our indica rice phosphoproteome, as the most extensive set of identified phosphosites in rice endosperm, greatly enriches the plant post-translational modification information. 

Phosphorylation-mediated regulation associated with high-temperature stress has been explored in rice leaves [35]. Researchers have found the dephosphorylation of ribulose bisphosphate carboxylase (RuBisCo) and the phosphorylation of ATP synthase subunit-β under heat stress [35]. However, the potential phosphorylation-mediated regulation remains to be disclosed owing to the limited data. A recent large-scale phosphoproteomic study of wheat leaf and spikelet revealed temperature-induced interconversion of neighboring phosphorylation residues [26]. So far, the phosphorylation-mediated potential in cereal endosperm under heat stress remains unexplored. This study is the first large-scale investigation to focus specifically on the phosphorylation status of rice endosperm under high-temperature stress.

### 3.1. An Essential Role of CDPKs against Heat Stress 

CDPKs belong to the CAMK kinase family and sense changes in the cytoplasmic Ca^2+^ concentration in response to abiotic stress and translate these perceived signals into subsequent downstream signaling events to trigger response mechanisms/pathways [36]. Among all differentially regulated kinases (Table S7), 9 CDPKs with 15 residues occurred most in all kinase subfamily (Figure 4b and Figure S15), and 14 phosphosites tended to be up-regulated in a high-temperature environment (Figure 4b), suggesting that CDPKs is likely a critical factor activated by high temperature. Similarly, this inference was supported by the result of phosphorylation motif analysis presented above that consensus motifs ([sP], [LxRxxs], [Rxxs], [tP]) induced by heat stress are potential substrates for CDPKs (Figure 4a). In other words, evidence from two aspects may provide novel insights for subsequent studies of CDPKs in response to heat stress.

In the literature, so far, kinase–substrate networks induced by high-temperature have not been investigated in detail [26]. Here, we focused on a potential regulatory network mediated by SPK, a kind of CDPK specifically expressed in immature endosperm. SPK showed increasing the phosphorylation intensity of three sites induced by heat stress, and two up-regulated phosphosites (S303, S317) were shared by both vaterites (Figure 4b and Figure S11b and Table S7). Based on this finding, we boldly speculated that the kinase activity of SPK is subsequently activated to deal with the possible deficiency in starch accumulation induced by heat stress. Using the approach of in vitro phosphorylation, Asano et al. found that a serine residue at the N-terminal region of sucrose synthase is a target of SPK [37]. However, it is still unclear which sucrose synthase isoform(s) is phosphorylated actually in vivo. In this study, four possible target phosphosites were screened from developing seeds for the first time: SUS2-S10, SUS3-S15, SUS4-S11, and SUS5-S12 (Figure S11b). Under high-temperature treatment, only SUS5-S12 in 9311 was significantly up-regulated as we expected, but the phosphorylation intensity of SUS2-S10 was down-regulated in GLA4 (Figure 5). In addition, no significant differences emerged in the phosphorylation intensity of SUS3-S15 and SUS4-S11. Two plausible reasons could explain those findings. One possibility is that SPK alone does not induce these phosphosites. Indeed, the phosphorylation status of the specific sites is likely a consequence of multiple protein kinases and phosphatases. Another potential explanation is that the protein expression of SUSs is severely inhibited by heat stress [7] so that the up-regulated phosphorylation is not sufficient to compensate for the loss of protein expression.

### 3.2. RNA Splicing Is a Critical Pathway in Response to Heat Stress

RNA splicing, a form of RNA processing, removes introns and joins exons together to make the pre-mRNA transform into a mature messenger RNA (mRNA). Recent observations have suggested that post-transcriptional regulation, especially alternative splicing (AS), appears to function in plant responses to environmental stress [38]. In our phosphoproteomic dataset, the spliceosome pathway was greatly over-represented in both varieties by KEGG pathway predication (Figure S5c). Phosphorylation events (430 phosphosites corresponding to 80 proteins) occurred within almost all spliceosome complexes (Figure S16), indicating that RNA splicing might be a critical pathway response to high-temperature stress in rice endosperm. 

Ser/Arg (SR)-rich proteins are a group of RNA-binding proteins that finely regulate alternative splicing by interacting with pre-mRNA sequences and splicing factors during spliceosome assembly [39]. Our work found a substantial number of phosphorylation sites on rice SR proteins (RSZp21a, RSZp21b, and RSZ23; Figure S17). In rice, a prior study noted the importance of SR protein in constitutive and alternative splicing of Pre-mRNA, and RSZp23 enhanced the splicing of the *Wx^b^* gene at the proximal sites [40]. It is worth mentioning that the RS domain of Arabidopsis RSZp22, homologous to rice RSZp23, regulates its shuttling between nucleoplasm and nucleolus through its level of phosphorylation [41,42]. Therefore, in the present study, we focused on the 12 phosphorylated serine sites of RSZp23 and found that nine phosphosites were located in the sequence of RSYSRSP at the RS domain (Figure S17). We considered whether there was a plausible mechanism by which the phosphorylation of the RS domain in RSZp23 might influence the splicing efficiency of *Wx^b^*. The indica variety 9311 carrying *Wx^b^* observed a down-regulated phosphorylation trend in RSZp23 induced by heat stress (Figure S18). In contrast, GLA4 exhibited up-regulation trends (Figure S18). Under high-temperature treatment (10 DAF), the GBSSI protein in 9311 slightly increased, whereas that of GLA4 declined (Figures S7 and S8). This inconsistency may be due to post-transcriptional regulation of RSZp23, which is likely a major factor affecting the alternative splicing of *Wx*. 

Previous studies have revealed the association between the splicing efficiency of *Waxy* and temperature in that high temperature caused a significant decrease in the number of mature mRNAs [43]. Overall, the results presented in this study provide valuable insights into the hypothesis that adjustment of the RSZp23 phosphorylation pattern induced by high temperature affects the splicing efficiency of *Waxy* and eventually influences amylose biosynthesis. In this sense, the potentially essential differences of SR proteins between 9311 and GLA4 at the phosphorylation level may provide novel ideas for the improvement of starch quality.

### 3.3. Phosphorylation Regulates Amylose Biosynthesis

Amylose content is one of the key factors that strongly influence rice grain quality. Regretfully, to date, few studies have been able to carry out any systematic research of amylose biosynthesis mediated by phosphorylation, particularly under abiotic stress. The potential roles of phosphorylation in regulating amylose biosynthesis were addressed in three dimensions (Figure 6c).

First, the phosphorylation of regulatory factors and splicing factors may affect the expression of GBSSI (Figure 6c). In rice endosperm, OsbZIP58 bonds directly to the promoters of multiple rice starch biosynthetic genes in vivo, including AGPL3, Wx, SSIIa, SBEI, BEIIb, and ISA2, which affects the accumulation of starch during grain development [44]. A mutant analysis reported that FLO2 alters the expression of various genes involved in sucrose and starch metabolism by mediating protein–protein interactions [45]. Although there is currently no evidence of the biological functions of phosphorylated regulatory factors, a large number of phosphosites detected in the present study still provide a possible connection between the gene expression of GBSSI and the phosphorylation status of those regulatory factors. In addition, as mentioned above, the phosphorylation status of RSZp23 may affect the splicing efficiency of *Wx^b^*, modulating GBSSI expression at the post-transcriptional level.

Second, the most crucial point to note is that 11 phosphosites of GBSSI were identified in GLA4 (Figure 6a). In rice endosperm, GBSSI is a key enzyme specifically responsible for elongating amylose polymers and serves as the only protein known to be required for amylose synthesis [46]. In recent years, Pro-Q Diamond dye and LC-MS/MS have been employed to determine the phosphosites of GBSSI in rice and wheat endosperm [47,48]. More specifically, Zhang et al. found at least nine phosphosites in rice GBSSI and inferred that the S415P substitution may modulate its enzyme activity regulated by phosphorylation levels [49]. As far as we know, our study is the most extensive-scale study of the GBSSI phosphorylation event. Sequence alignment analyses indicated that the six residues are phosphorylated at the same position that corresponds to japonica rice and wheat (Figure 6a) [48,49]. Remarkably, all significantly up-regulated phosphosites (S123, S169, and S553) are conserved among plant GBSSI, suggesting their functional importance. Despite these promising results, further data collection is required to determine precisely how heat affects the phosphorylation level of GBSSI and subsequently influences amylose synthesis.

Third, protein targeting starch (PTST) was phosphorylated in rice endosperm (Figure 6c). PTST is originally a carbohydrate-binding scaffold protein that interacts with GBSSI as a pre-requisite for subsequent starch granule binding in Arabidopsis [50]. Zhong et al. carried out a series of experiments and confirmed the vital importance of PTST for amylose biosynthesis in maize endosperm [51]. Our study revealed two novel phosphosites (S213 and S289) in indica rice endosperm. The phosphorylation of S289 was uniquely detected at 9311-H. Similarly, the intensity of GLA4-H was 3.71-fold in comparison with GLA4-C, although this difference did not reach statistical significance (Figure 5). It is thus likely that kinases targeting this phosphosite might be activated by high temperature and eventually affects the localization of GBSSI. 

### 3.4. Phosphorylation Regulates Amylopectin Biosynthesis

Amylopectin is the major component of starch and serves as a key substance in rice grains. Several works on maize and wheat endosperm have emphasized the vital importance of protein phosphorylation in the formation of a starch-synthesizing protein complex [10,11]. Dephosphorylation of BEs and SSs is associated with reduced activity and protein complex formation [10,11,12,52]. In rice endosperm, although several investigations observed the importance of high-molecular-weight complexes for maintaining the activities of corresponding key enzymes [53], the phosphorylation-dependent formation of the complexes remains unknown [54]. In this sense, sequence alignment analysis was carried out to obtain the information about the similarities and differences between rice and other plants.

SS, covering four isoforms, SSI, SSII, SSIII, and SSIV, has been attributed to catalyze the chain elongation reaction of α-1,4-glucosidic linkages in amylopectin synthesis [55]. Analysis of cereal endosperm appears to show that each isoform of SS performs a specific role in amylopectin synthesis [10]. Consistent with the review by Croft et al., sequence alignment analysis revealed that approximately all SSIIa phosphosites identified are not conserved among various plant species [19]. It has been demonstrated that wheat starch synthesis enzymes SSI and SSIIa are phosphorylated [11,48] and the protein complex including SSII is phosphorylation dependent [10,11]. However, relevant evidence in rice SSIIa is still lacking. Nakamura et al. concluded that SSIIa activity is a determinant affecting thermal gelatinization properties [46]. Gelatinization temperature witnessed a clear trend of significantly increasing in 9311 under heat stress (Table S1). Interestingly, the phosphorylation intensity of a single peptide of SSIIa was only detected in 9311-C (Figure 5), suggesting that enzyme activity and protein complex formation are likely to be determined by the phosphorylation status rather than the protein abundance. 

BEs are responsible for catalyzing the formation of α-1,6-glucosidic linkages of amylopectin in rice endosperm [55]. The phosphorylation sites of BEs with different conservations may display variations in protein structure, binding location, and kinase specificity [19]. To probe such possibility in plant BEs, we, thus, performed sequence alignment analyses of BEs from eight plants (rice, maize, barley, wheat, potato, sorghum, pea, and Arabidopsis) and focused on novel identified phosphorylation sites (Figure 6b). As is apparent, some phosphosites were conserved among all plant BEs, while others were species specific (Figure 6b). In particular, some residues of OsBEIIb (S323/S324, S625), OsBEIIa (S466/467), and OsBEI (S562) were phosphorylated at the same alignment positions when compared with maize (Figure 6b) [15,56,57,58] and wheat (Figure 6b) BEs [59,60]. Moreover, a close link was observed in three BE isoforms in that they possessed overlapping phosphosites (Figure 6b). In the present case, plant BEs possess the most conserved phosphorylation pattern among all starch-synthesis-related enzymes, suggesting a high probability of the conserved regulation mechanism in cereal endosperm.

Western blot analysis of five residues of rice BEs (BEI-S562, BEI-S620, BEI-S821, BEIIb-S685, and BEIIb-S715) further verified that the degree of phosphorylation change is possibly significantly associated with corresponding site conservation (Figure 7e–i and Table S12). In other words, high-temperature treatment tends to have a greater effect on non-conserved phosphosites. This phenomenon can also be confirmed in phosphoproteomic analysis in that heat stress triggered phosphorylation at S11 and S173 of BEIIb but exerted suppressive effects on BEIIa-S467. Only the conserved phosphosite BEIIb-T580 located at GH13 was an exception, showing an up-regulation trend under high-temperature stress in 9311 groups. Overall, our findings still support the hypothesis that BEI-S562 and BEIIb-S685, as conserved sites in the functional domain, reflect steady-state phosphorylation levels during heat stress to maintain the relative stability of essential functions. As non-conserved sites that may play specific roles, BEI-S620, BEI-S821, and BEIIb-S715 might exhibit more flexible phosphorylation regulation patterns when exposed to an adverse environment. These findings contribute to a better understanding of phosphorylation-mediated regulation under abiotic stress and provide a solid basis for subsequent preparation of corresponding mutants to verify the specific function in the near future.

DBE consists of isoamylase (ISA) and Pullulanase (PUL) with catalytic function for hydrolyzing α-1,6-glucosic linkages [55]. Recent experiments have shown that the function of PUL appeared to be positively regulated by high temperature [61]. However, a postulated and legitimately interpreted mechanism for this phenomenon remains absent [61]. Our present study aims to fill this gap at the phosphorylation level. As shown in Figure 5, the phosphorylation intensity of S221 was significantly enhanced. It is thus most likely that the phosphosite (PUL-S221) within the functional domain is regulated by potential kinases and subsequently affects the enzymatic activity of the domain.

Plastidial phosphorylase (Pho1), a temperature-dependent enzyme, is considered crucial not only during the maturation of amylopectin but also in the initiation process of starch synthesis [19]. Satoh et al. examined the effects of low temperature on the *pho1* mutant and found an essential role of Pho1 during its initial stages of α-glucan biogenesis, especially under conditions of low temperature [62]. Hence, they speculated that one or more other factors are involved to replace Pho1 in a high-temperature environment. Most phosphosites are conserved among monocotyledonous plants, and five phosphosites (S341, S932, S939, S944, T974) explicitly localized at the phosphorylase domain are highly conserved among all plants (Figure S10). In particular, S341 was identified as an active site pocket of glycogen phosphorylase and similar proteins (cd04300; InterPro), suggesting a link may exist between phosphorylation and functional activity. An early report has demonstrated that the serine residue in the L80 region from the sweet potato root is phosphorylated [63], and we found three phosphosites in the same region, although their alignment positions were not completely consistent (Figure S10). Under high-temperature stress, the phosphorylation intensity of T376 exhibited a reverse trend between two varieties (Figure 5). These phosphosites identified in rice Pho1 with significant differences under high-temperature stress are expected to become a new breakthrough point for functional activity research.

## 4. Materials and Methods

### 4.1. Plant Materials and Experimental Design

Two indica rice varieties (*Oryza sativa* L.) 9311 and Guangluai4 (GLA4) were used to examine the effect of high temperature on the developing rice endosperm with different starch qualities. The detailed parameters of starch qualities are provided by Pang et al. [22]. All plant materials were planted at the experimental farm of Zhejiang University under normal rice cultivation conditions until heading. Before the flowering stage, all rice plants were transferred into artificial climatic chambers at a suitable temperature (20–28 °C) with a 14/10 h photoperiod (day/night). Each panicle, on the day of rice flowering, was tagged to facilitate collecting samples at defined developmental stages. Five days later, rice plants of the treatment group were moved into a high-temperature chamber (HT; 30–38 °C; Figure S1) and then exposed to high temperature for 1, 5, and 9 days. In the meantime, other plants were still cultivated at a suitable temperature as controls (CT; 20–28 °C; Figure S1). All temperature treatments were completed under other consistent growth conditions, and the relevant parameters were as follows: the illumination time was 14 h/day (5:00–19:00), the illumination intensity during the light phase was 10,000–12,000 Lux, the relative humidity was 70–80%, the maximum temperature difference was 8 °C, and the average wind speed was 0.5 m/s. During grain development, rice panicles were handpicked from fresh plants at different periods (6, 10, and 14 DAF) in three separate biological replicates and immediately frozen in liquid nitrogen (9311/GLA4-C, 9311/GLA4-H; Figure 1a). For phosphorylation studies, developing seeds collected at 10 DAF were first determined by LC-MS/MS and then all samples in three periods were analyzed by Western blot using site-specific phosphopeptide antibodies. In addition, the remaining seeds were harvested at maturity for chalkiness degree and starch quality analysis (9311/GLA4-C0, 9311/GLA4-H1, 9311/GLA4-H3, 9311/GLA4-H5; Figure 1a). 

### 4.2. Determination of Starch Quality

Under flowing nitrogen conditions, differential scanning calorimetry (DSC) measurements were conducted using a TA instrument Q20 (TA Instruments, New Castle, DE, USA) at a heating rate of 10 °C /min according to the method described by Bao et al. [64]. The onset temperature (To), peak temperature (Tp), conclusion temperature (Tc), gelatinization enthalpy (ΔH) and width at half peak height (ΔT_1/2_) were obtained from the DSC thermogram. Three replicates were performed per sample. Duncan’s multiple-range test of ANOVA was performed in SPSS (IBM SPSS Statistic 20). Statistical significance was defined at the level of *p* < 0.05.

### 4.3. Protein Preparation, Digestion, and Phosphopeptide Enrichment

For the extraction of endosperm proteins, other tissues (husk, pericarp, and embryo) were removed from immature rice grains [53]. The procedures were quickly carried out on ice. Rice endosperm was then homogenized by grinding in the presence of liquid nitrogen prior to protein extraction. In brief, rice endosperm was extracted with extraction buffer (4% SDS, 1mM DTT, 100 mM Tris–HCl, pH 7.6) supplemented with EDTA-free protease and phosphatase inhibitor cocktails (Sigma-Aldrich, St. Louis, MO, USA). Sonication was performed using 10 rounds of 10 s sonication and 3 s off-sonication. Following boiling for 10 min, protein samples were centrifuged at 12,000× *g* for 40 min at 4 °C. Supernatants were gathered and kept in a freezer at −80 °C for subsequent phosphoproteomic pretreatment. The protein amount was estimated by BCA assay (Pierce BCA Protein assay kit, Thermo Fisher Scientific, Waltham, MA, USA).

For FASP digestion, samples were treated as previously described [65]. SDS, DTT, and other low-molecular-weight components were removed using UA buffer (8M Urea, 150 mM Tris-HCl, pH 8.0) by repeated ultrafiltration (10 kDa, Satorious, Gottingen, Germany). Then protein mixtures were alkylated with 100 mM iodoacetamide (IAA) for 30 min in darkness. After repeated washing with UA buffer and 25 mM NH_4_HCO_3_, the protein suspensions were digested with trypsin (Promega, Madison, WI, USA) overnight at 37 °C. The enzymatic peptides were desalted on a Sep-Pak C18 cartridge (Waters, Milford, MA, USA) and subjected to TiO_2_-based phosphopeptide enrichment, as previously described by Pang et al. [22]. The enriched eluates of each sample were concentrated by vacuum evaporation and reconstituted in 0.1% formic acid for MS analysis.

### 4.4. LC-MS/MS and Data Analysis

Liquid chromatography–tandem mass spectrometry (LC-MS/MS) analysis was performed using an Easy-nLC System coupled with a Q-Exactive Plus mass spectrometer (Thermo Fisher Scientific, Waltham, MA, USA). The mobile phases consisted of 0.1% formic acid (A) and 0.1% formic acid in 84% *v/v* acetonitrile (B). The column was equilibrated with 95% solution A. A volume of 6 μL of phosphopeptide solution was loaded onto Acclaim PepMap100 (100 μm × 2 cm, nanoViper C18, 3 μm, 100 Å; Thermo Fisher Scientific, Waltham, MA, USA), and separated by an EASY column (10 cm, ID75 μm, 3 μm, C18-A2; Thermo Fisher Scientific, Waltham, MA, USA) at a flow rate of 300 nL/min. Over a period of 0–2 min, the concentration of solution B rose linearly from 5% to 7%; from 2 to 162 min, it increased from 7% to 25%; from 162 to 225 min, it increased from 25% to 40%; from 225 to 230 min, it increased from 40% to 100%; and from 230 to 240 min, it was maintained at 100%. 

The Q-Exactive Plus mass spectrometer was operated in positive ion mode over 240 min. Full-scan mass spectra were acquired over a mass range of 300–1800 *m*/*z*. The resolution of first-order mass spectrometry was 70,000, the AGC target was 1 × 10^6^, and the first-order maximum IT was 50 ms. For subsequent MS2 analysis, only the top 10 precursors were selected. HCD-MS2 spectra were acquired with 1 microscan at a resolution of 17,500, and the AGC target was 1 × 10^5^. The MS2 scan range was set from 200 to 2000 *m*/*z*, the maximum IT was 110 ms, and the isolation window was 2.0 *m*/*z*. Dynamic exclusion was employed with an exclusion duration of 60 s. Three biological replicates were performed independently for each group (Figure S2a).

Raw mass spectrometric data were processed with MaxQuant software (version 1.5.5.1) and compared with the indica rice protein sequence database (*Oryza sativa* subsp. *indica*-ASM465v1). Trypsin/P was specified as the enzyme, and two missed cleavages were allowed. The precursor mass tolerance was set to 20 ppm for the first search (used for mass re-calibration) and to 4.5 ppm for the main search. The MS/MS mass tolerance was set to 20 ppm. Carbamidomethylation of cysteine residues was selected as a fixed modification, while protein N-terminal acetylation, methionine oxidation, and phosphorylation on serine/threonine/tyrosine were allowed as variable modifications. False discovery rate (FDR) thresholds for protein, peptide, and modification sites were specified at 1%. A minimum peptide length of seven amino acids was required. The mass spectrometry proteomics data have been deposited to the ProteomeXchange Consortium (http://proteomecentral.proteomexchange.org, accessed on 2 July 2021) via the iProX partner repository [66] with the dataset identifier PXD027052 and the subject ID IPX0003230000. Groups N1, N2, L1, and L2 in the PXD027052 project correspond to groups 9311-C, 9311-H, GLA4-C, and GLA4-H in this study, respectively.

### 4.5. Statistical and Bioinformatic Analyses

Quantification of the modified peptides was performed using the label-free quantification algorithm. In general, phosphosites that exhibited valid values in one condition (at least 2 of 3 replicates) and none in the other indicate a massive change in phosphorylation levels. We, therefore, opted to select those phosphosites that feature a consistent presence/absence profile in 9311 and GLA4, respectively. On the rest of the dataset, we performed Student’s t-test (*p* < 0.05, log_2_ (fold-change) > 1) on phosphosites with at least two valid values in any condition. For a more comprehensive understanding of phosphosites showing significant differences in expression, phosphorylation sites that were uniquely identified in either condition and significantly regulated from the statistical test were combined and divided into four groups (9311-Up, 9311-Down, GLA4-Up, and GLA4-Down; Figure 3d). 

A total of 13 amino acid (AA) sequences centered by the phosphosite were extracted, and the enriched phosphorylation motifs induced by high-temperature stress were predicted using the MoMo tool (http://meme-suite.org/tools/Momo, accessed on 9 January 2020) with the motif-x algorithm. The conserved motif patterns were then redrawn and visualized by TBtools [67]. Heatmaps for the relative abundances, Venn diagrams, and upset plots for various lists were also produced using TBtools. GO enrichment analysis was conducted using the AgriGO website (http://bioinfo.cau.edu.cn/agriGO/, accessed on 6 January 2020). Cytoscape (http://www.cytoscape.org, accessed on 6 January 2020) was used to generate network visuals. KEGG pathway annotation was performed by using KEGG Automatic Annotation Server (KAAS) software (http://www.genome.jp/kegg/kaas, accessed on 18 December 2019). A *p*-value of <0.05 was used as the threshold of significant enrichment. Pfam (www.sanger.ac.uk/Software/Pfam/, accessed on 12 February 2020) and InterPro (https://www.ebi.ac.uk/interpro/, accessed on 12 February 2020) were used to identify functional domains. Protein sequences were aligned using T-coffee. The figures were annotated with Adobe Illustrator (Adobe Systems, San Jose, CA, USA). 

### 4.6. Preparation of Primary Antibodies

Abundantly conventional and site-specific phosphopeptide antibodies were used for this experiment. 

Conventional antibodies: Anti-rice GBSSI, SSIIIa, BEI, and BEIIb antibodies) were kindly gifted by Prof. Naoko Fujita (Akita Prefectural University, Akita, Japan). Mouse monoclonal antibodies against plant actin were obtained from Sigma-Aldrich (Sigma-Aldrich, St. Louis, MO, USA). 

Site-specific phosphopeptide antibodies: Modification peptides of interest from phosphoproteome were selected and are listed in Table S11. The synthetic phosphorylated peptides were coupled to keyhole limpet hemocyanin (KLH) before immunization of rabbits. The antibodies were purified from rabbit polyclonal antiserum by affinity purification via sequential chromatography on phosphopeptide and non-phospho-peptide affinity columns (Affinity Biosciences, Changzhou, China). The efficiency of antibody production was monitored using ELISA. 

### 4.7. Western Blotting

Protein expression of starch-synthesis-related enzymes as well as the site-specific phosphorylation intensity were examined by Western blot assay [53]. Total proteins were separated by SDS-PAGE after loading buffer was added. Immediately, separated proteins were transferred to a PVDF membrane (Merck Millipore, Billerica, MA, USA) using a Trans-Blot Cell system (Bio-Rad Laboratories, Hercules, CA, USA). The membranes proceeded directly to the blocking step and then were exposed to appropriate antibodies. Chemiluminescence signals were developed with an ECL kit (SuperSignal West pico; Thermo Fisher Scientific, Waltham, MA, USA) and detected by a ChemiDoc imaging system (Bio-Rad Laboratories, Hercules, CA, USA). Correspondingly, analysis of bands was performed with Image Lab™ software (Bio-Rad Laboratories). For normalization, within the same membrane, plant actin was used as a loading control. For evaluating the relative phosphorylation intensity, phosphorylated phosphosites were corrected to the levels of the corresponding total protein. Statistical analysis was performed using ANOVA with Tukey’s post-test in GraphPad Prism (GraphPad Software, San Diego, CA, USA).

## 5. Conclusions

In conclusion, this study set out to provide the first systematic investigation of the phosphoproteome induced by high-temperature stress in rice endosperm. Comparative analysis of the temperature-induced phosphorylation status revealed some interesting similarities and differences between two indica rice varieties (9311 and GLA4). On the one hand, both 9311 and GLA4 shared several consensus motifs ([sP], [LxRxxs], [Rxxs], [tP]) and were highly enriched in terms of heat response (GO) and the spliceosome pathway (KEGG). On the other hand, a dramatic difference was observed in the phosphorylation status of kinases induced by heat stress, which could reasonably explain the different phosphorylation regulatory patterns of 9311 and GLA4. More importantly, we detailed the most comprehensive starch metabolism pathway at the phosphorylation level in rice endosperm, including starch-synthesis-related enzymes (AGPase, GBSSI, SSIIa, SSIIIa, BEI, BEIIb, ISA1, PUL, PHO1, PTST), transcription factors (OsbZIP58 and FLO2), SR protein (RSZp23), and CDPK kinase (SPK). Western blot with site-specific phosphor-antibodies was used to verify and explore the dynamic change of the phosphorylation status of SSIIIa (SSIIIa-S94), BEI (BEI-Ser562, BEI-Ser620, BEI-Ser821), and BEIIb (BEIIb-Ser685, BEIIb-Ser715), which might play specific roles in amylopectin biosynthesis in response to high-temperature stress. An exciting phenomenon was discovered in that conserved sites tend to reflect steady-state phosphorylation levels. However, non-conserved sites exhibit more flexible phosphorylation regulation patterns when exposed to high-temperature stress. These findings provide valuable insights into the role of phosphorylation response to high-temperature stress, adding unprecedented depth and breadth to the cereal research community.

## Data Availability

The mass spectrometry proteomics data have been deposited to the ProteomeXchange Consortium (http://proteomecentral.proteomexchange.org, accessed on 2 July 2021) via the iProX partner repository [66] with the dataset identifier PXD027052 and the subject ID IPX0003230000 (https://www.iprox.cn/page/project.html?id=IPX0003230000, accessed on 2 July 2021).

## Supplementary Materials

The following are available online at https://www.mdpi.com/article/10.3390/ijms221910546/s1.

## Abbreviations

AGPase	ADP-glucose pyrophosphorylase
AMY	α-amylase
BAM	β-amylase
BE	Starch branching enzyme
DAF	Days after flowering
DBE	Starch debranching enzyme
DSC	Differential scanning calorimetry
FK	Fructokinase
GBSS	Granule-bound starch synthase
ΔH	Enthalpy of gelatinization
HK	Hexokinase
INV	Invertase
ISA	Isoamylase
PGI	Glucose-6-phosphate isomerase
PGM	Phosphoglucomutase
PHO1	Plastidial phosphorylase
PTM	Post-translational modification
PUL	Pullulanase
SPP	Sucrose-phosphate phosphatase
SPS	Sucrose-phosphate synthase
SS	Starch synthase
SUS	Sucrose synthase
SUT	Sucrose transporter
Tc	Conclusion temperature
To	Onset temperature
Tp	Peak temperature
UDPG	UDP-glucose
UGPase	UDP-glucose pyrophosphorylase
