# Supplementary material for: Comparative Phosphoproteomic Analysis Reveals the Response of Starch Metabolism to High-Temperature Stress in Rice Endosperm
Source: Int J Mol Sci. 2021 Sep 29;22(19):10546. doi: 10.3390/ijms221910546 (PMC8508931; doi:10.3390/ijms221910546)

Supplementary Figures

Comparative phosphoproteomic analysis reveals the response of starch metabolism to high-temperature stress in rice endosperm

Yuehan Pang[1], Yaqi Hu[1] and Jinsong Bao[1*]

1 Institute of Nuclear Agricultural Sciences, College of Agriculture and Biotechnology, Zhejiang University, Zijingang Campus, Hangzhou, 310058, China

* Correspondence: jsbao@zju.edu.cn

Figure S1. Temperature variation of control and high-temperature treatment groups in artificial climatic chambers.

Figure S2. (a) Experimental design for the phosphoproteome of rice endosperm in response to heat stress. (b) Mass error distribution of all identified peptides. (c) Peptide length distribution. (d) Heatmap of the Pearson correlation among the samples from different biological replicates.

Figure S3. Upset plots and Venn diagrams of significantly differentially phosphosites (a) and phosphoproteins (b) in 9311-C,9311-H, GLA4-C, and GLA4-H.

Figure S4. (a) The percentage of kinases detected in our study and the rice protein database. Significantly enriched kinase families in phosphoproteome were labeled with an asterisk ($P<0.05$). (b) Summary of kinases with significant differences in response to heat stress. Venn diagram at left compares the identified kinases between the two varieties, whereas the right depicting the overlap of significantly differentially phosphosites in 9311-C,9311-H, GLA4-C, and GLA4-H.

Figure S5. Enrichment analysis based on gene ontology (GO) terms and KEGG pathway.

(a-b) GO biological process (GO-BP) and GO molecular function (GO-MF) enrichment analysis. Enrichment networks from four significantly deregulated groups were visualized using Cytoscape plugins Enrichment Map with different colors. Each node represents an enriched GO term, and the node size corresponds to the number of phosphoproteins. Edge thickness shows the overlap of GO terms between neighbor nodes. (c) KEGG enrichment analysis of differentially phosphorylated proteins of 9311 and GLA4.

Figure S6. Transcription factors identified in phosphoproteome.

Figure S7. Western blot assay of GBSSI at the three grain-filling stages. Western blots were performed three independent times, and the best blot was shown. The results of the independent experiments were following a similar trend in expression. Uncropped western

blots are available in Figure S20.

Figure S8. Evaluation of the protein level expression of starch synthesis related enzymes at the three grain-filling stages. The relative expression was normalized using plant actin. *P<0.05, **P<0.01, ***p<0.001, ****p<0.0001

Figure S9. Domain structure of SSIIa and SSIIIa and their phosphosites with significant differences in response to heat stress.

Figure S10. Domain structure and amino acid sequence alignments of PHO1. Residues in yellow indicate the phosphorylation site. Non-phosphorylated residues are shown in dark grey.

Figure S11. (a) Regulatory relationships between phosphorylated regulatory factors and their target genes in rice endosperm. Blue and red arrows indicate activation and suppression, respectively. The black dotted line represents homologous regulators of other cereal crops. (b) Potential target phosphosites of SPK in sucrose synthase. Residues in orange indicate the phosphorylation site. Non-phosphorylated residues are shown in dark grey.

Figure S12. Mass spectrometry of synthetic phosphorylated peptides.

Figure S13. Blocking peptide experiments show the specificity of the phosphor antibodies. Immunolabeling was virtually eliminated by phosphorylated peptide blocking. PBP, phosphorylated peptide blocking; NBP, non-phosphorylated peptide blocking.

Figure S14. Phosphorylation levels of SSIIIa-S94, BEI-S562, BEI-S620, BEI-S821, BEIIb-S685, and BEIIb-S715 detected by LC-MS/MS and western blot at 10 DAF. The intensity of LC-MS/MS was obtained from Table S2. The relative expression by western blot was normalized using plant actin, and the phosphorylation levels presented here were not corrected by corresponding total protein for a more intuitive comparison with LC-MS/MS results.

Figure S15. The number of phosphosites (phosphoproteins) detected in each kinase subfamily.

Figure S16. Spliceosome pathway by KEGG. The orange shading represents phosphoproteins identified.

Figure S17. Domain structure and amino acid sequence alignments of RSZp21a, RSZp21b, and RSZp23. Residues in orange indicate the phosphorylation site. Non-phosphorylated residues are shown in dark grey.

Figure S18. Heatmap of normalized phosphorylation intensity and relative expression foldchange of SR proteins (RSZp21a, RSZp21b, and RSZp23) under high-temperature stress.

Figure S19. Uncropped western blots of Figure 7. Red boxes in the uncropped blots indicate the cropped regions shown in the corresponding Figures.

Figure S20. Uncropped western blots of Figure S7. Red boxes in the uncropped blots indicate the cropped regions shown in the corresponding Figures.

Figure S1

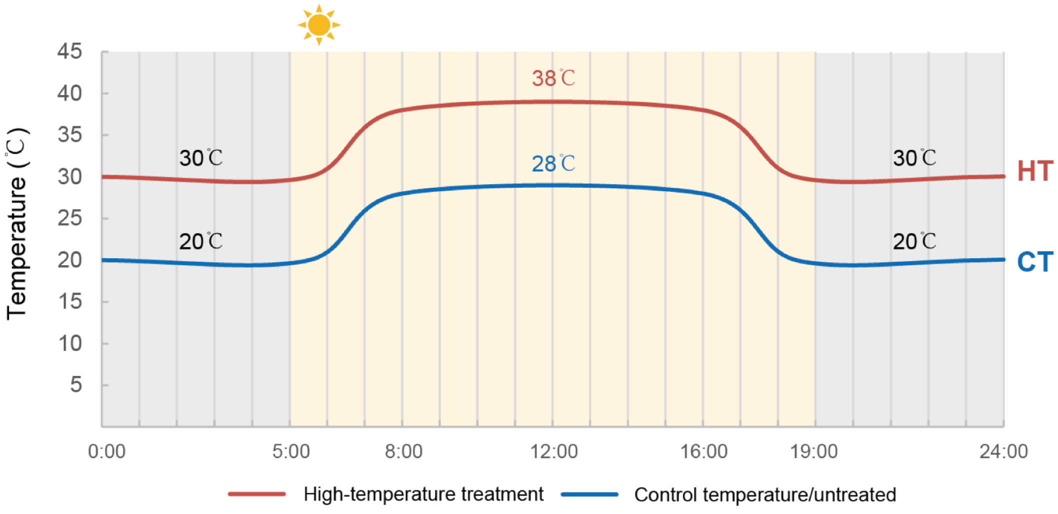

Figure S2

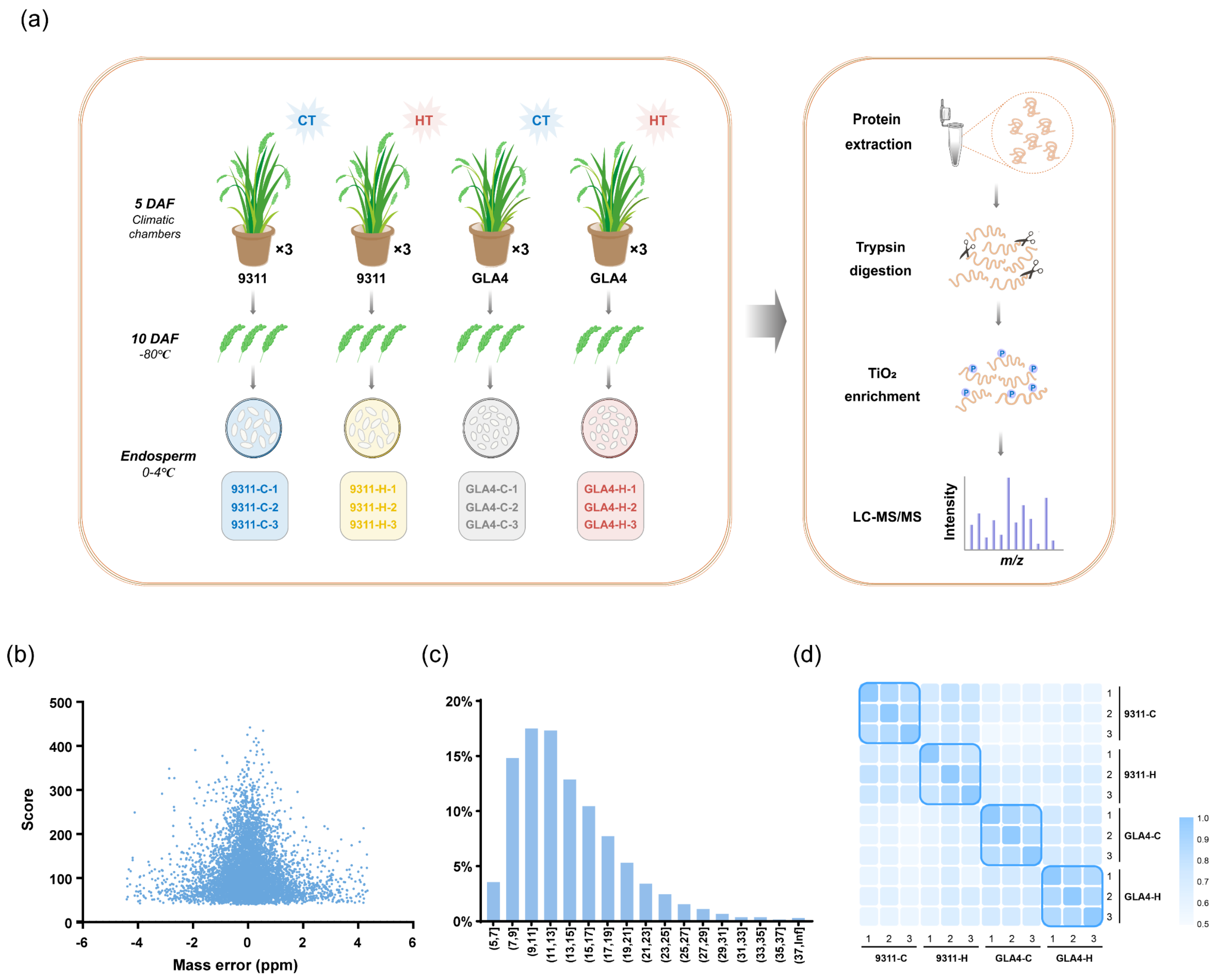

Figure S3

(a)

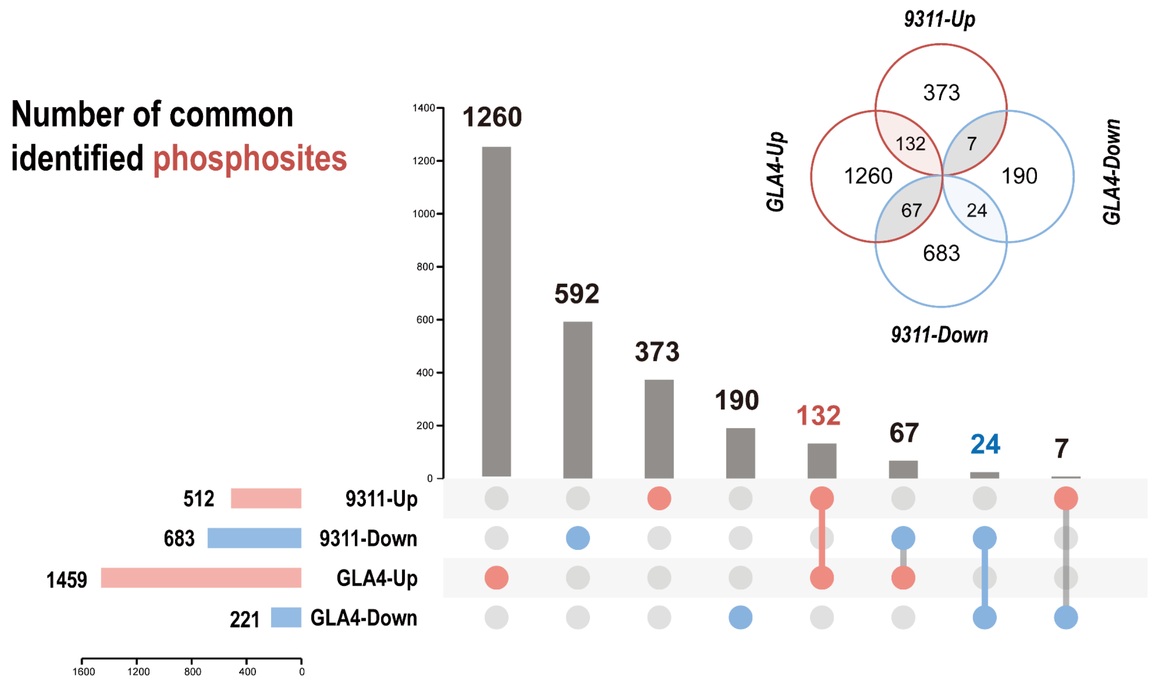

Number of common identified phosphosites
1260
592
373
190
132
67
24
7
512 9311-Up
683 9311-Down
1459 GLA4-Up
221 GLA4-Down
9311-Up
GLA4-Up
GLA4-Down
9311-Down
373
132
7
1260
190
67
24
683

(b)

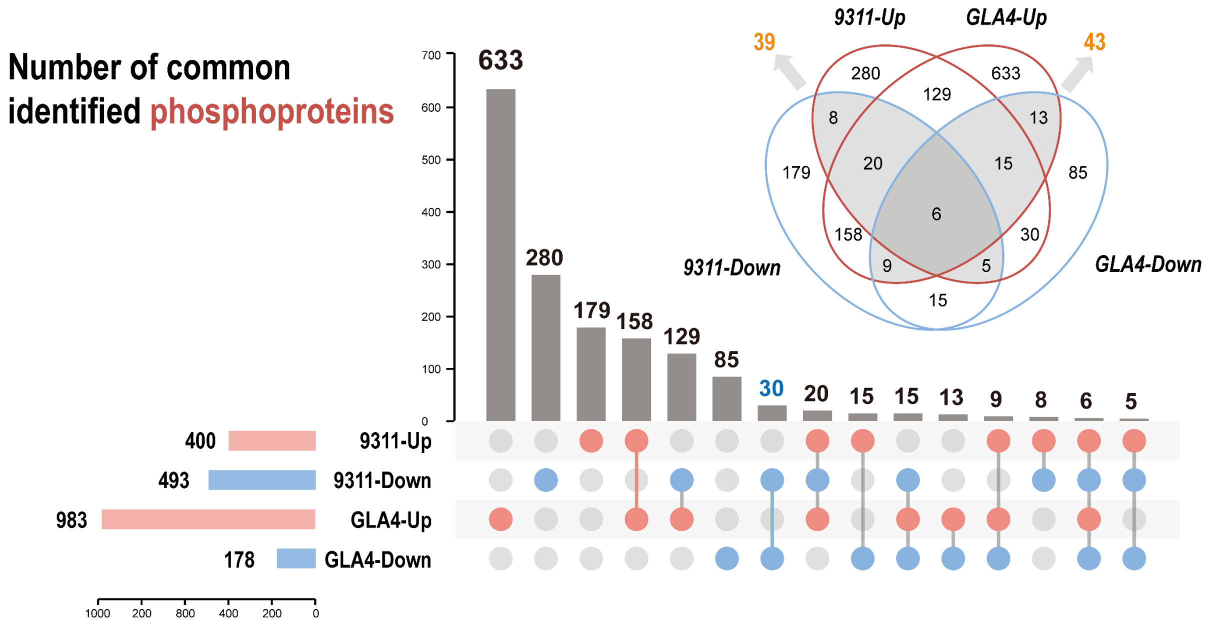

Number of common identified phosphoproteins
633
280
179
158
129
85
30
20
15
15
13
9
8
6
5
400 9311-Up
493 9311-Down
983 GLA4-Up
178 GLA4-Down
9311-Up
GLA4-Up
39
43
280
633
129
8
13
179
20
15
85
6
158
30
9
5
15
9311-Down
GLA4-Down

Figure S4

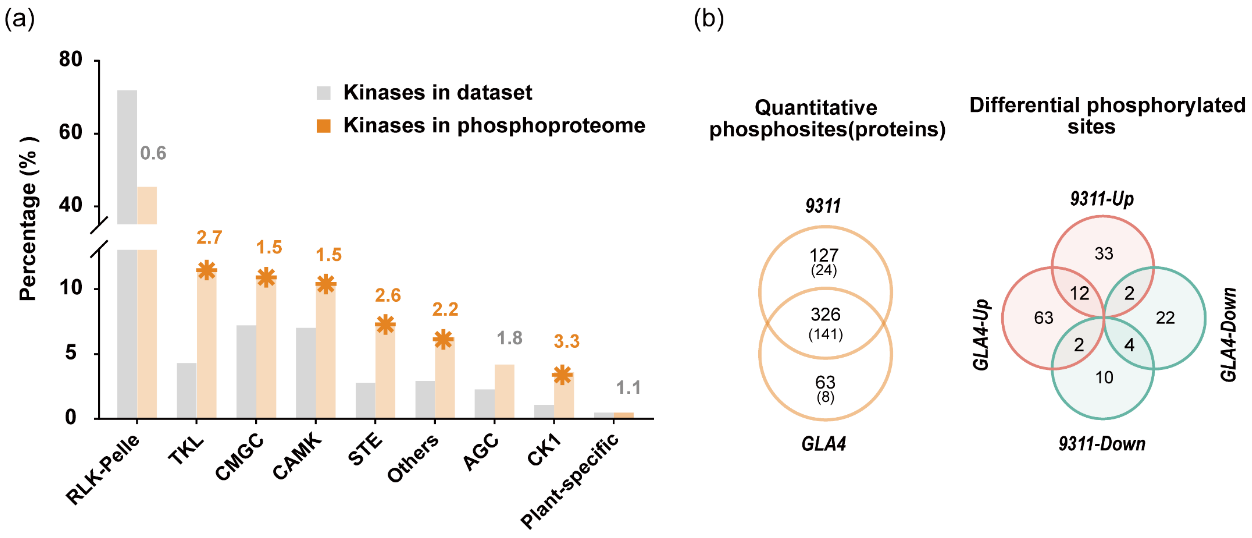

(a)
Percentage (%)
80
60
40
10
5
0
Kinases in dataset
Kinases in phosphoproteome
0.6
2.7
1.5
1.5
2.6
2.2
1.8
3.3
1.1
RLK-Pelle
TKL
CMGC
CAMK
STE
Others
AGC
CK1
Plant-specific
(b)
Quantitative phosphosites(proteins)
9311
127
(24)
326
(141)
63
(8)
GLA4
Differential phosphorylated sites
9311-Up
33
12
2
63
22
2
4
10
GLA4-Up
GLA4-Down
9311-Down

Figure S5

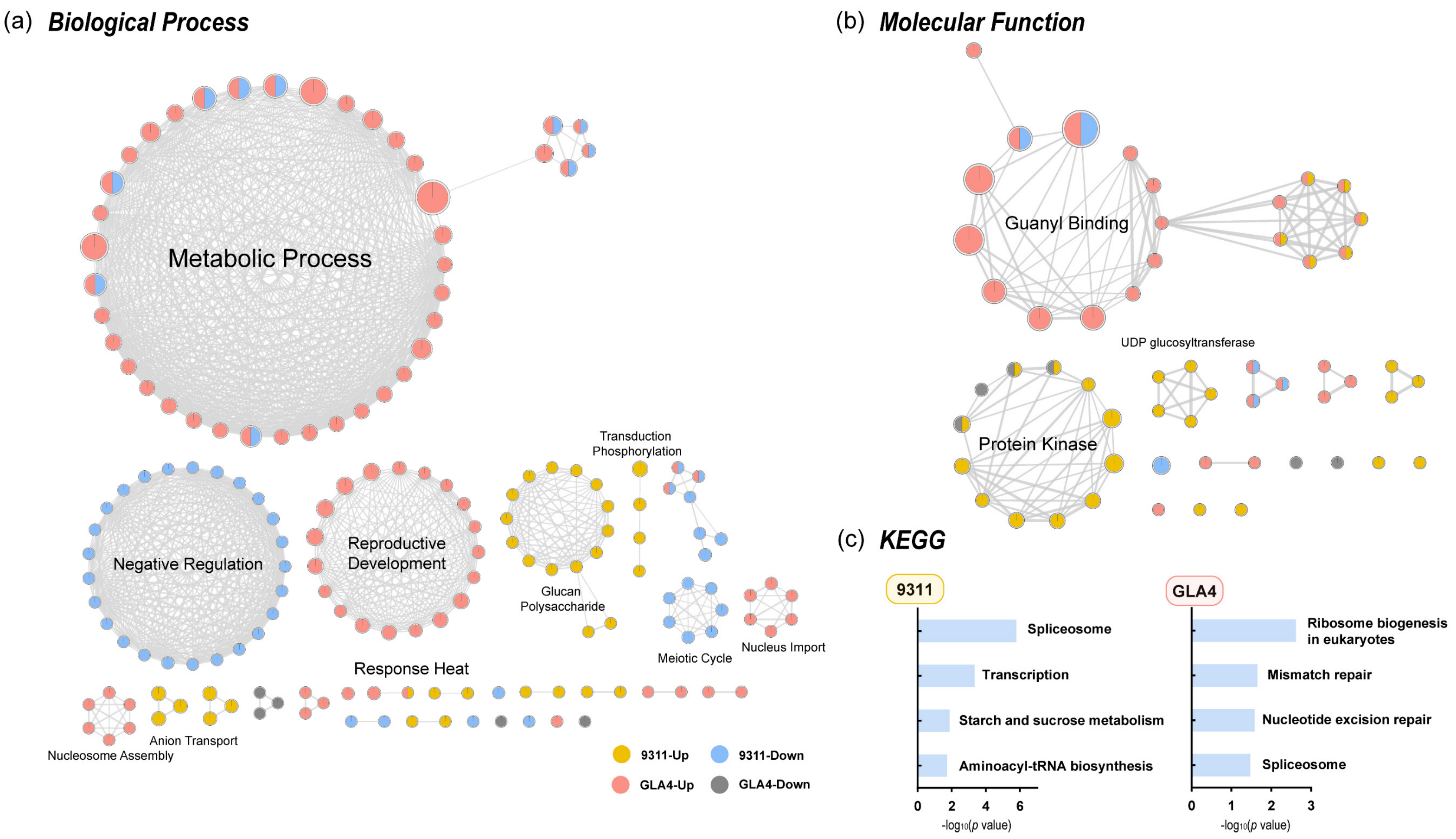
(a) Biological Process
Metabolic Process
Negative Regulation
Reproductive Development
Transduction Phosphorylation
Glucan Polysaccharide
Meiotic Cycle
Nucleus Import
Response Heat
Anion Transport
Nucleosome Assembly
9311-Up
9311-Down
GLA4-Up
GLA4-Down
(b) Molecular Function
Guanyl Binding
UDP glucosyltransferase
Protein Kinase
(c) KEGG
9311
Spliceosome
Transcription
Starch and sucrose metabolism
Aminoacyl-tRNA biosynthesis
0
2
4
6
-log10(p value)
GLA4
Ribosome biogenesis in eukaryotes
Mismatch repair
Nucleotide excision repair
Spliceosome
0
1
2
3
-log10(p value)

Figure S6

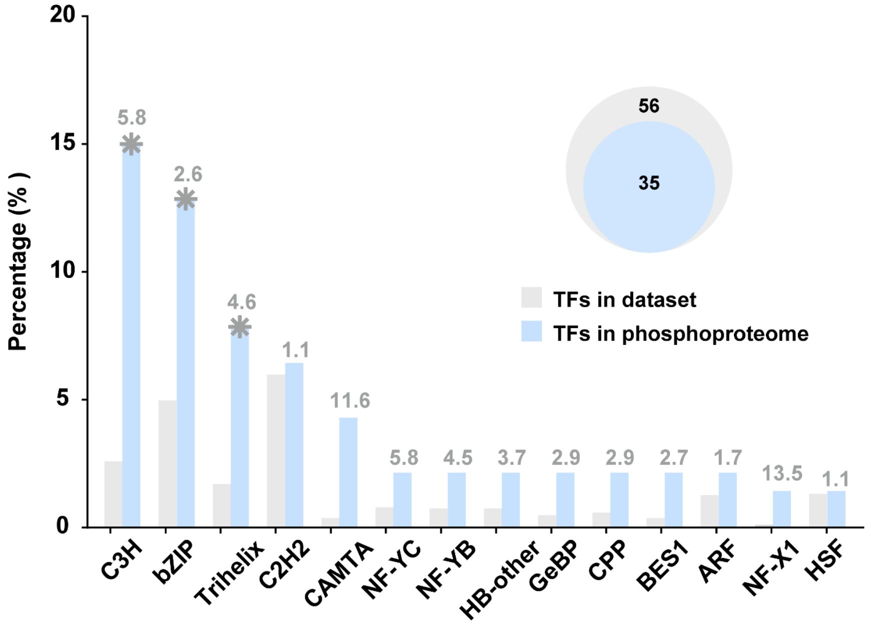

Percentage (%)
20
15
10
5
0
5.8
2.6
4.6
1.1
11.6
5.8
4.5
3.7
2.9
2.9
2.7
1.7
13.5
1.1
C3H
bZIP
Trihelix
C2H2
CAMTA
NF-YC
NF-YB
HB-other
GeBP
CPP
BES1
ARF
NF-X1
HSF
56
35
TFs in dataset
TFs in phosphoproteome

Figure S7

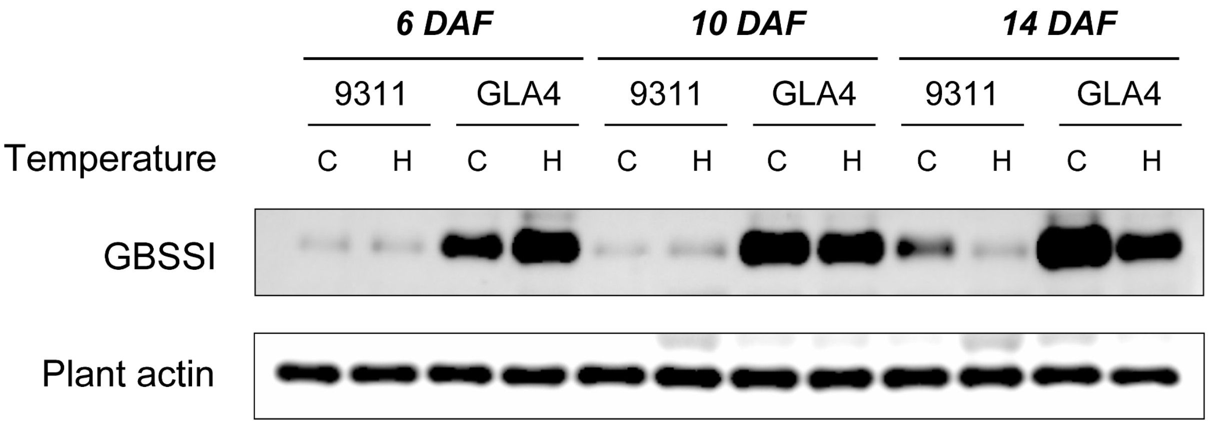

Figure S8

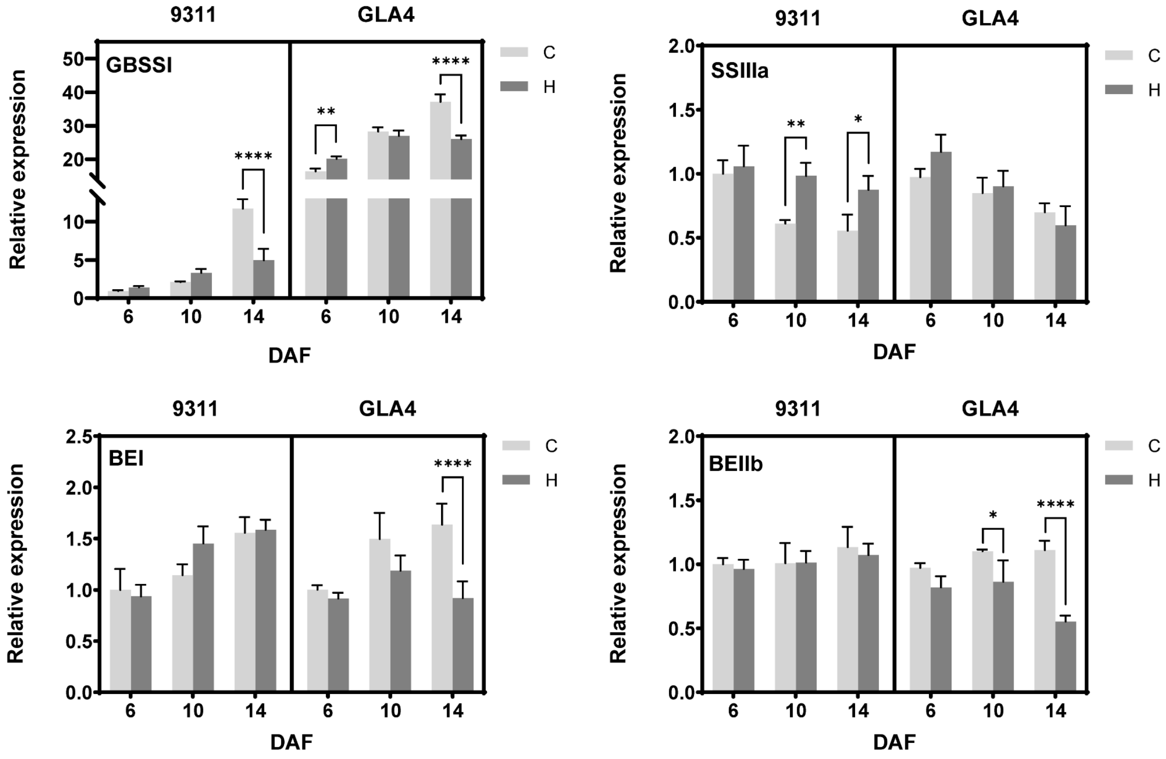

Figure S9

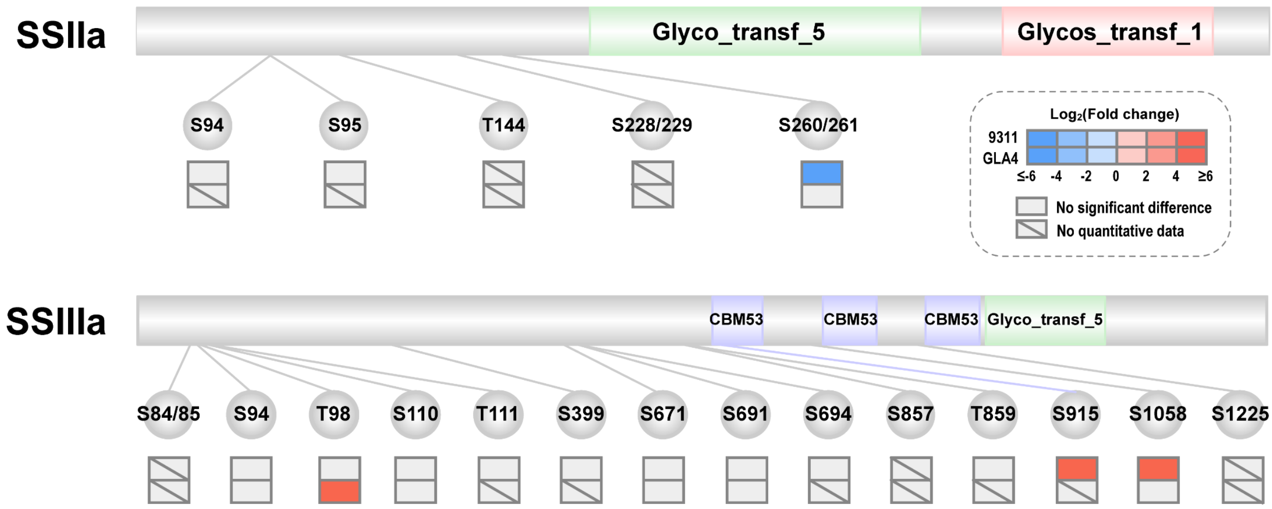

Figure S10

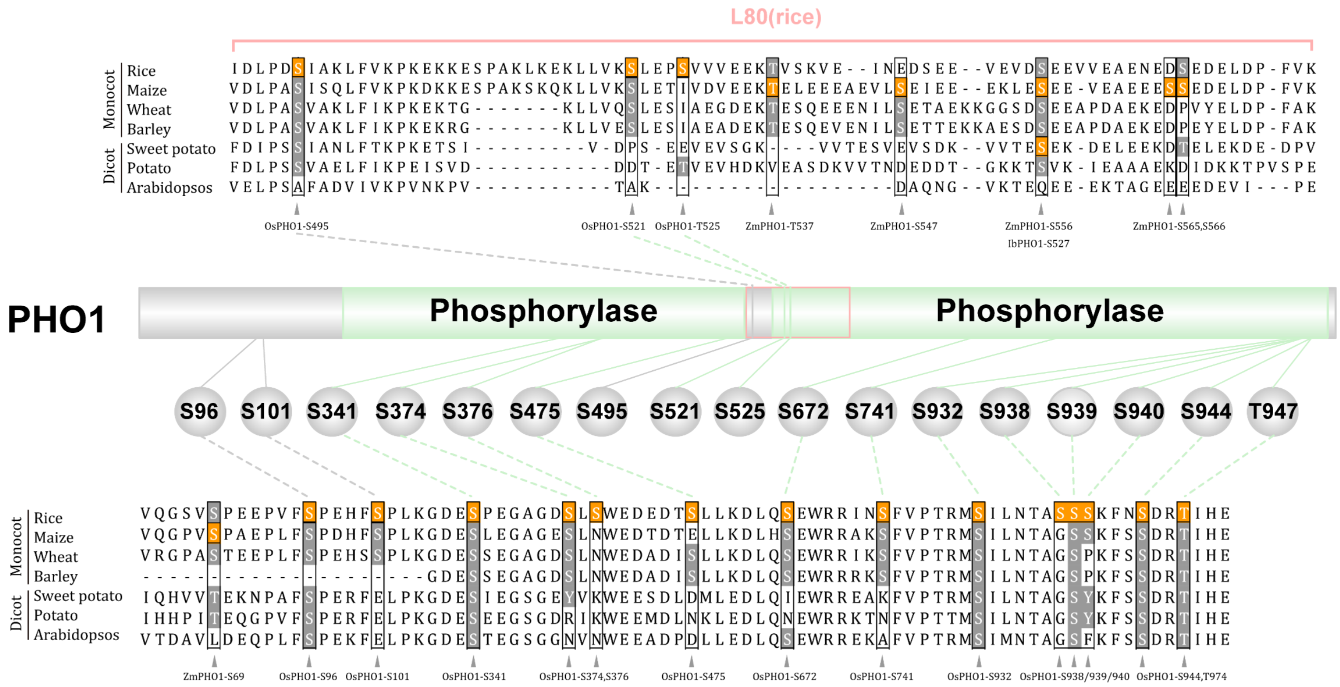

Figure S11

(a)

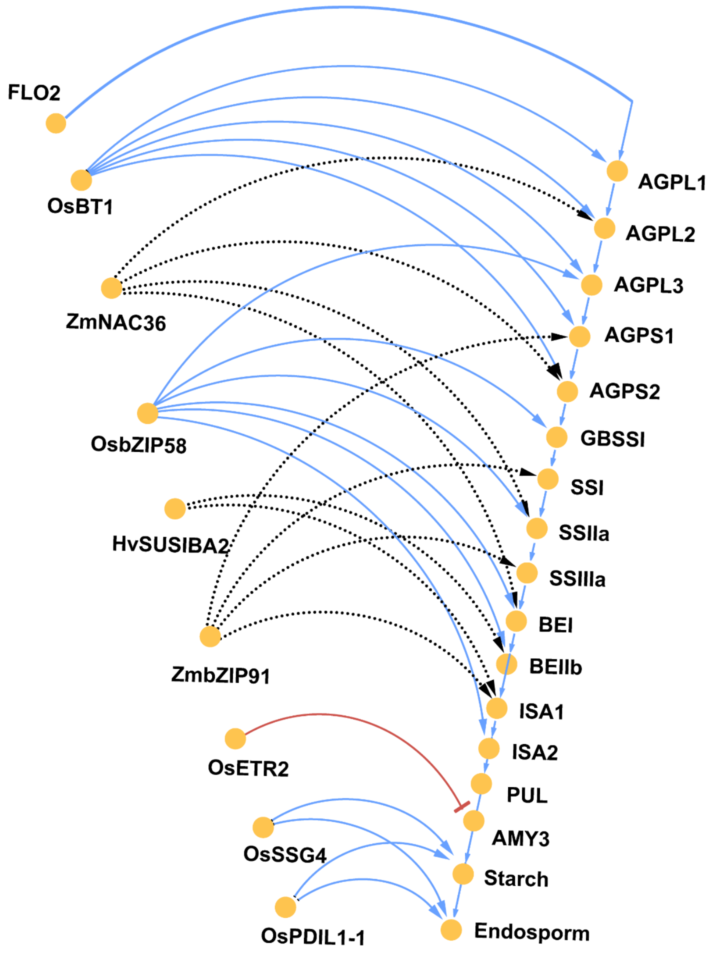

(b)

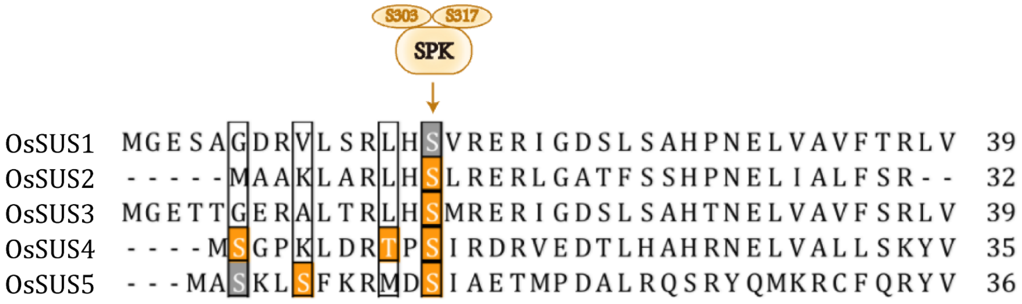

Figure S12

BEI-Ser562

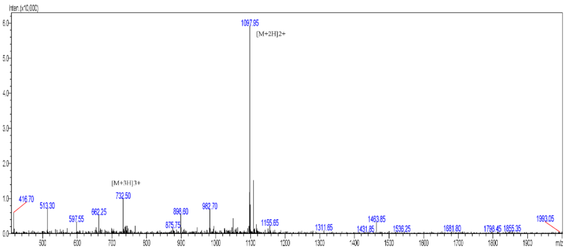

Phosphopeptide: EMYTGMSDLQPAS(ph)PTINR

M.W. : 2194.35 Da

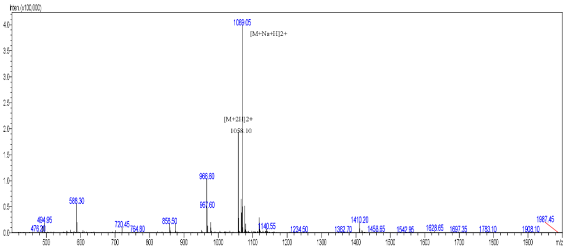

Non-phosphopeptide: EMYTGMSDLQPASPTINR

M.W. : 2114.37 Da

BEI-Ser620

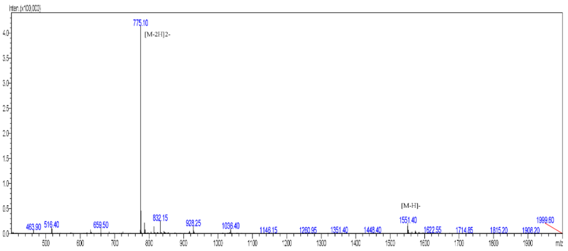

Phosphopeptide: QWS(ph)LVDTDHLR

M.W. : 1552.60 Da

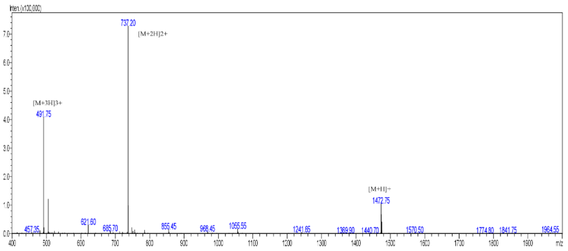

Non-phosphopeptide: QWSLVDTDHLR

M.W. : 1472.62 Da

BEI-Ser821

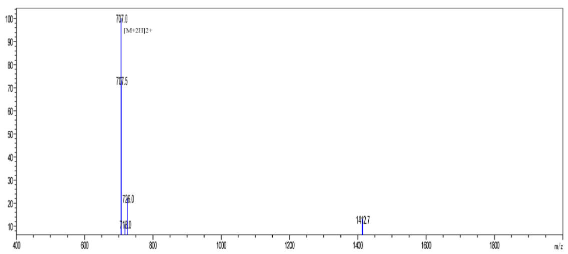

Phosphopeptide: FVFRS(ph)SDEDCK

M.W. : 1412.41 Da

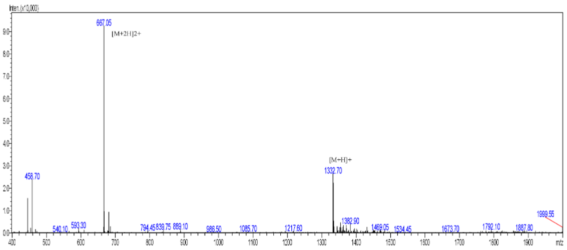

Phosphopeptide: FVFRSSDEDCK

M.W. : 1332.43 Da

BEIIb-Ser685

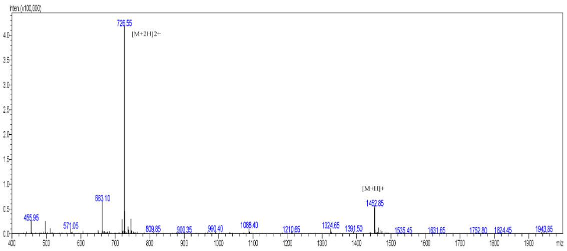

Phosphopeptide： FIPGNNNS(ph)YDK

M.W. : 1451.45 Da

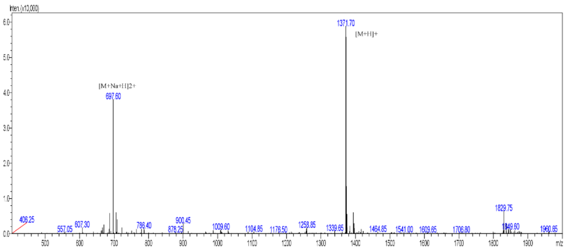

Non-phosphopeptide： FIPGNNNSYDK

M.W. : 1371.47 Da

BEIIb-Ser715

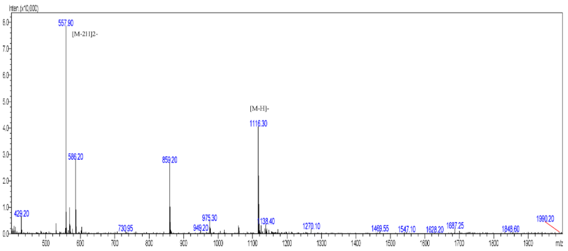

Phosphopeptide： AMQS(ph)LEEK

M.W. : 1118.17 Da

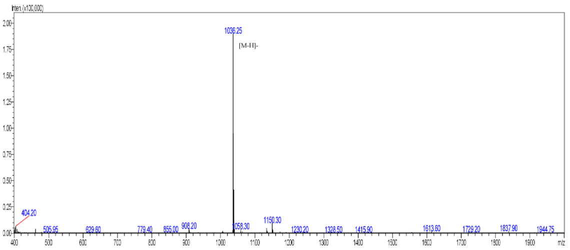

Non-phosphopeptide： AMQSLEEK

M.W. : 1038.19 Da

Figure S13

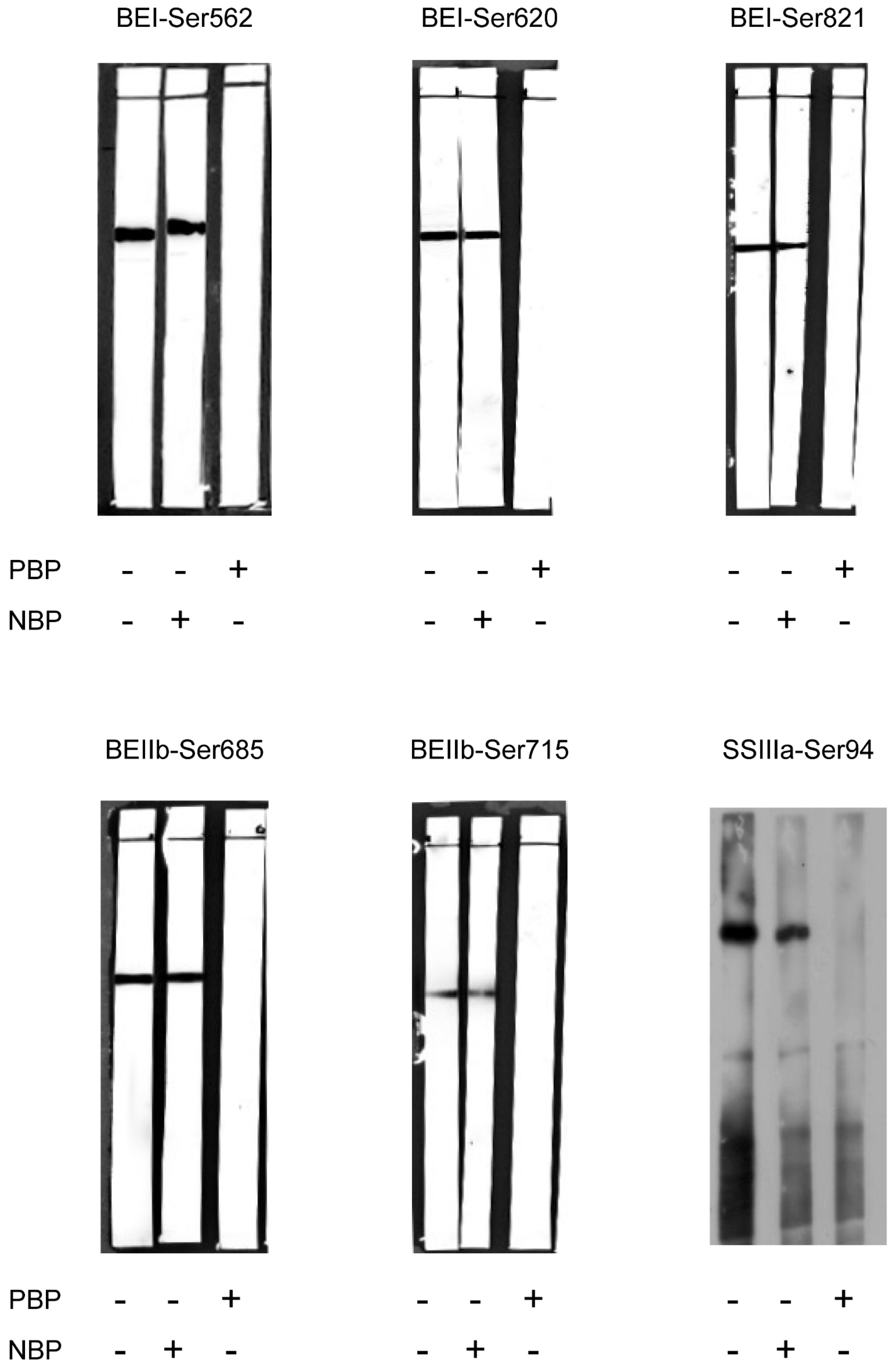

BEI-Ser562
BEI-Ser620
BEI-Ser821
PBP - - + - - + - - +
NBP - + - - + - - + -
BEIIb-Ser685
BEIIb-Ser715
SSIIIa-Ser94
PBP - - + - - + - - +
NBP - + - - + - - + -

Figure S14

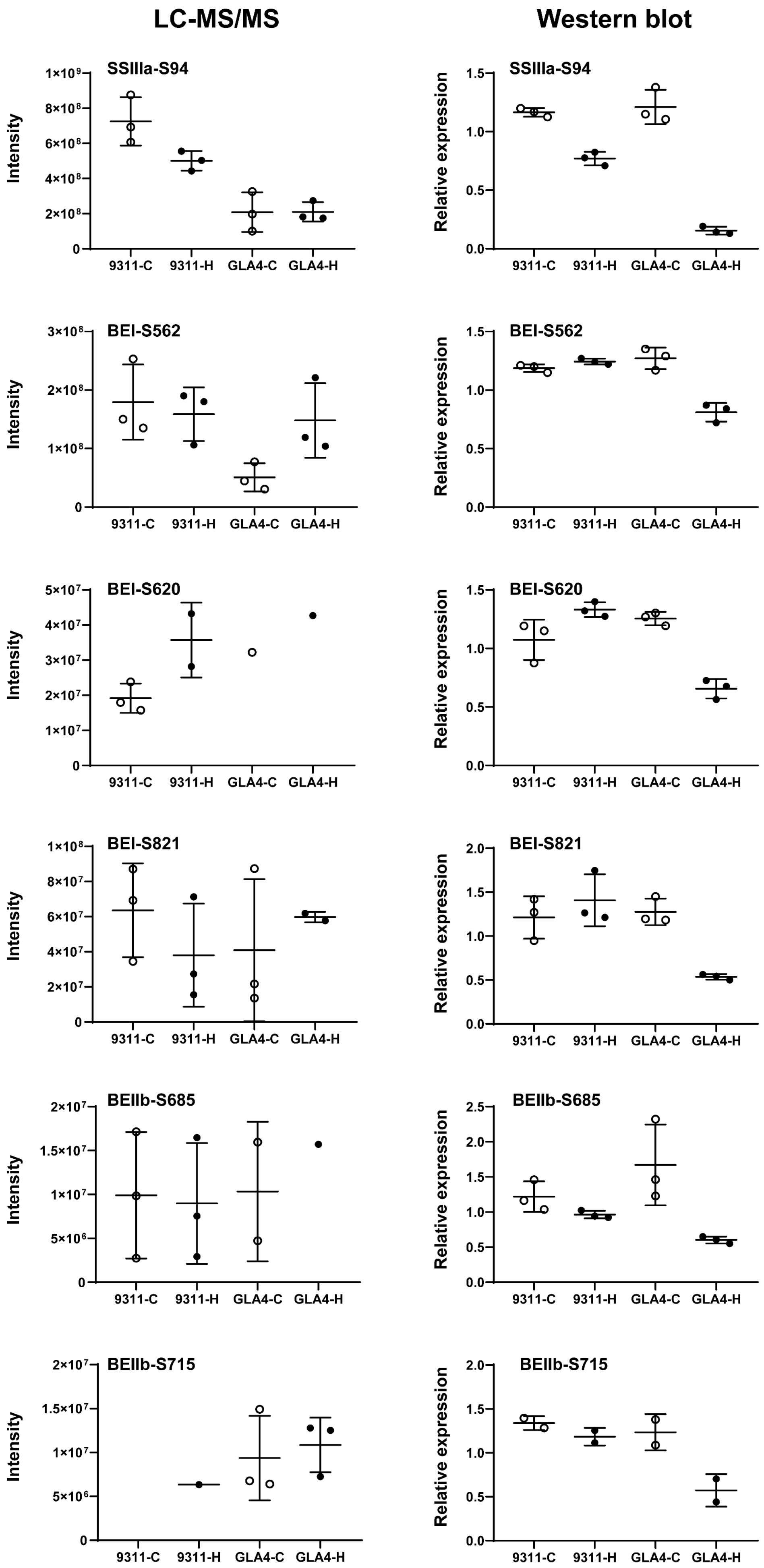

LC-MS/MS
Western blot
SSIIIa-S94
BEI-S562
BEI-S620
BEI-S821
BEIIb-S685
BEIIb-S715
Intensity
Relative expression
9311-C
9311-H
GLA4-C
GLA4-H

Figure S15

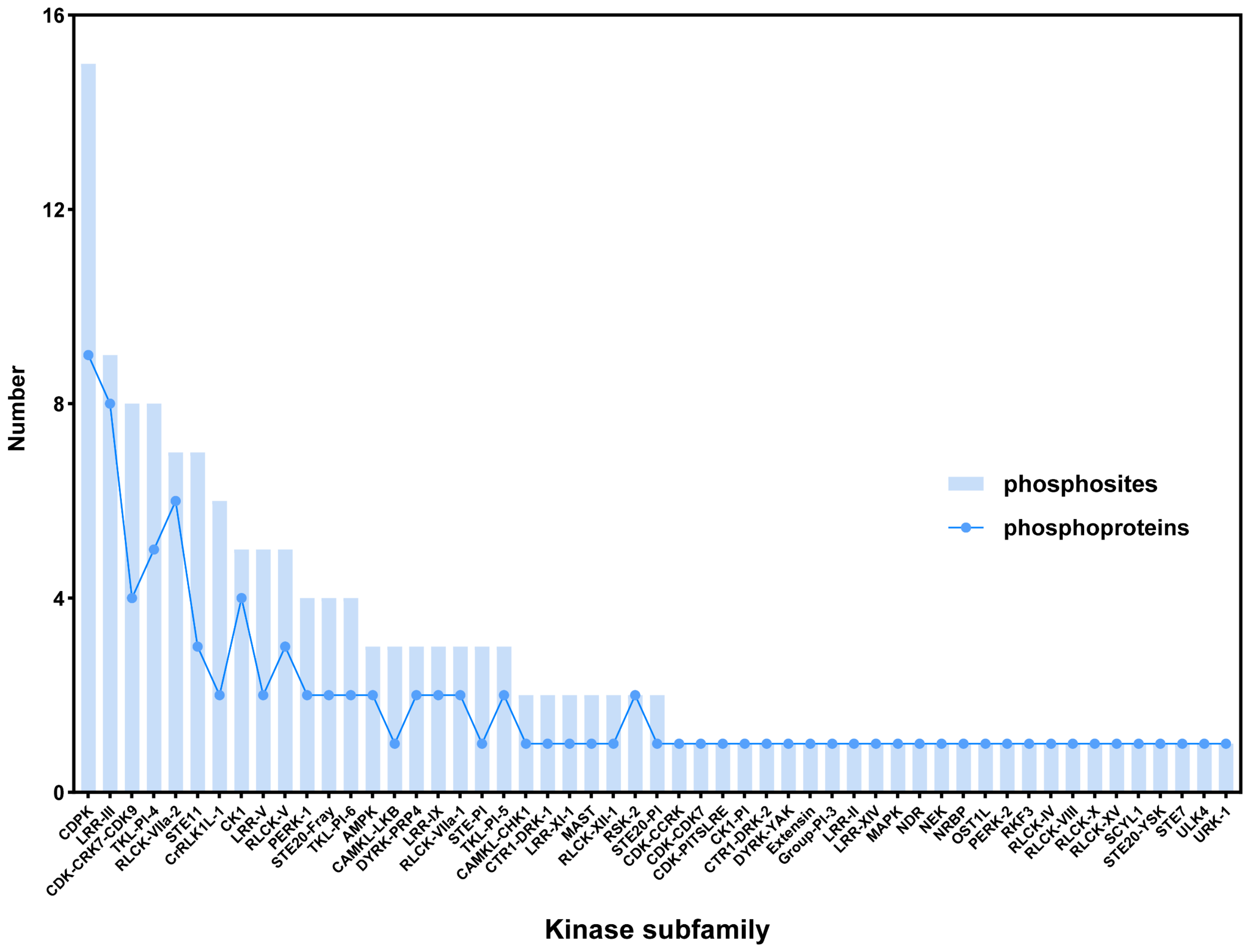

Number
0
4
8
12
16
phosphosites
phosphoproteins
CDPK
LRR-III
CDK-CRK7-CDK9
TKL-Pl-4
RLCK-VIIa-2
STE11
CrRLK1L-1
CK1
LRR-V
RLCK-V
PERK-1
STE20-Fray
TKL-Pl-6
AMPK
CAMKL-LKB
DYRK-PRP4
LRR-IX
RLCK-VIIa-1
STE-Pl
TKL-Pl-5
CAMKL-CHK1
CTR1-DRK-1
LRR-XI-1
MAST
RLCK-XII-1
RSK-2
STE20-Pl
CDK-CCRK
CDK-CDK7
CDK-PITSLRE
CK1-Pl
CTR1-DRK-2
DYRK-YAK
Extensin
Group-Pl-3
LRR-II
LRR-XIV
MAPK
NDR
NEK
NRBP
OST1L
PERK-2
RKF3
RLCK-IV
RLCK-VIII
RLCK-X
RLCK-XV
SCYL1
STE20-YSK
STE7
ULK4
URK-1
Kinase subfamily

Figure S16

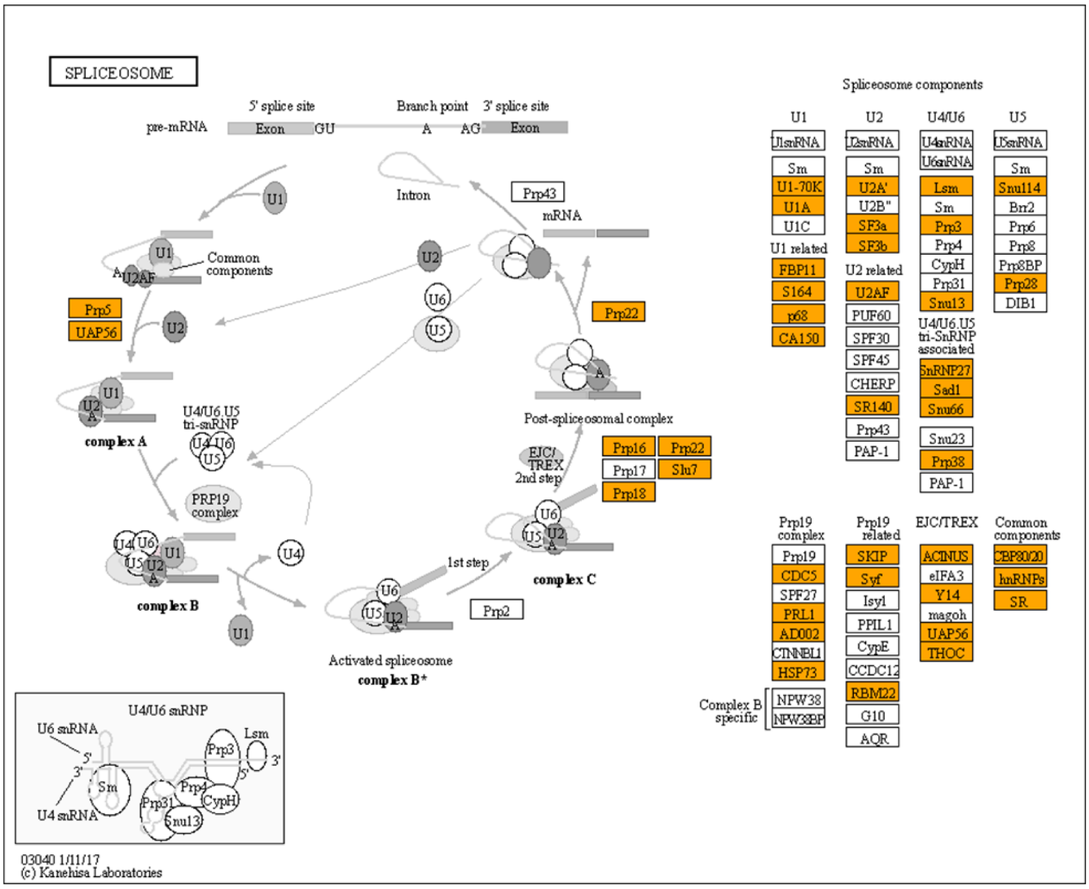

Figure S17

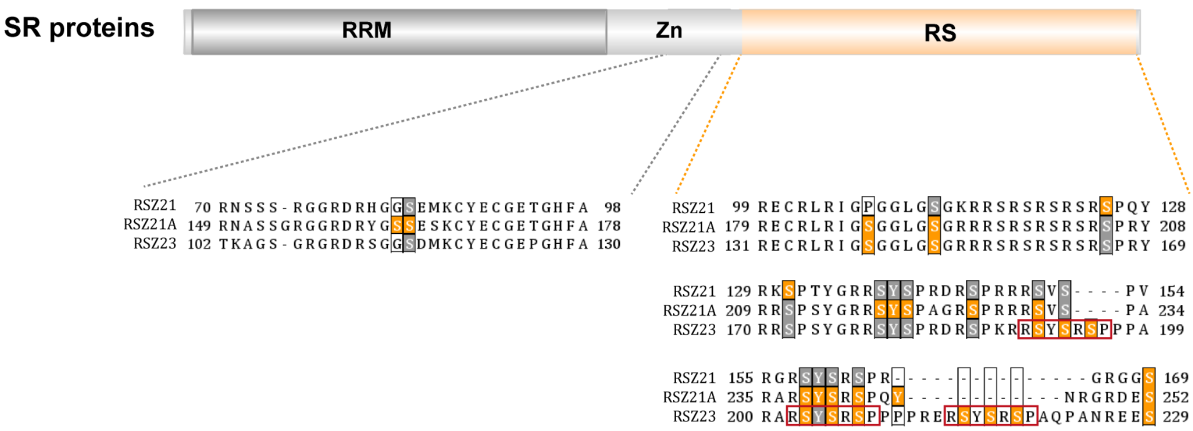

Figure S18

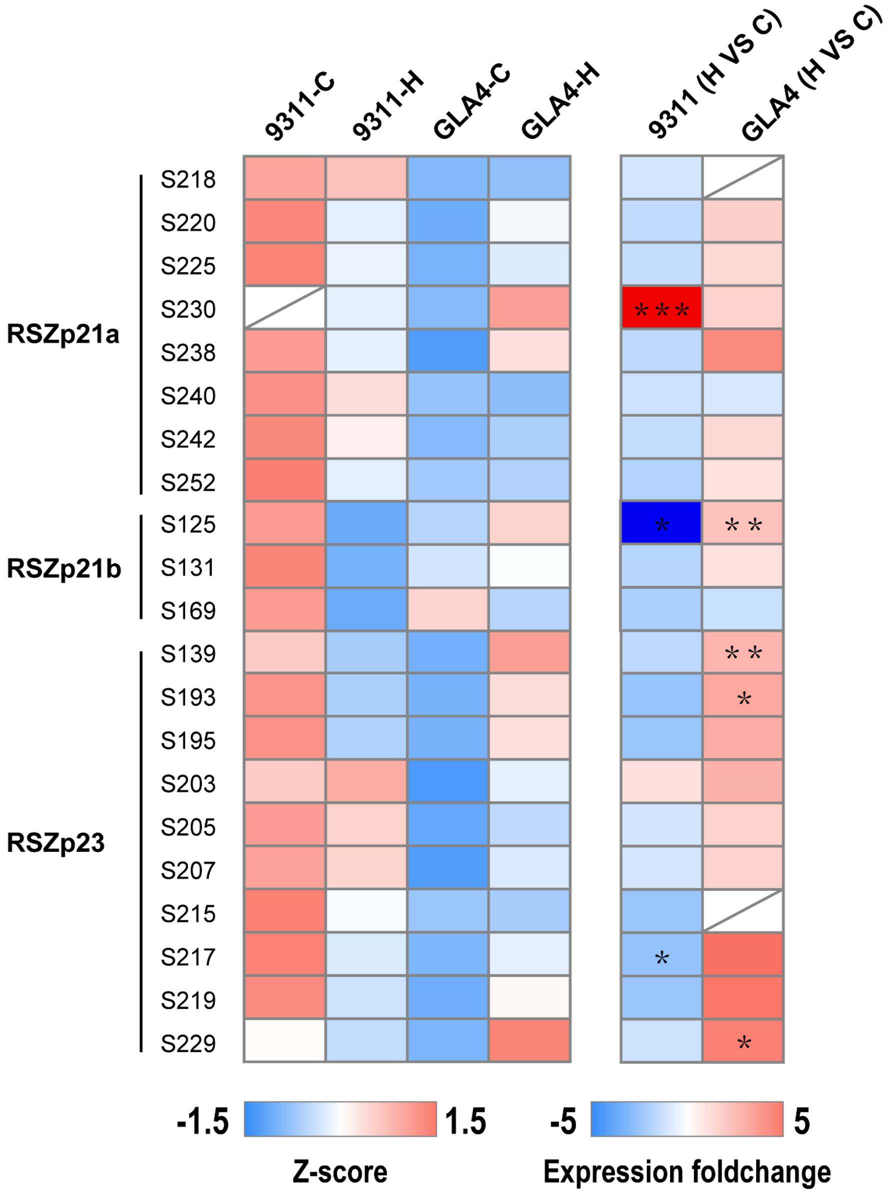

9311-C
9311-H
GLA4-C
GLA4-H
9311 (H VS C)
GLA4 (H VS C)
RSZp21a
RSZp21b
RSZp23
S218
S220
S225
S230
S238
S240
S242
S252
S125
S131
S169
S139
S193
S195
S203
S205
S207
S215
S217
S219
S229
-1.5
1.5
Z-score
-5
5
Expression foldchange

Figure S19

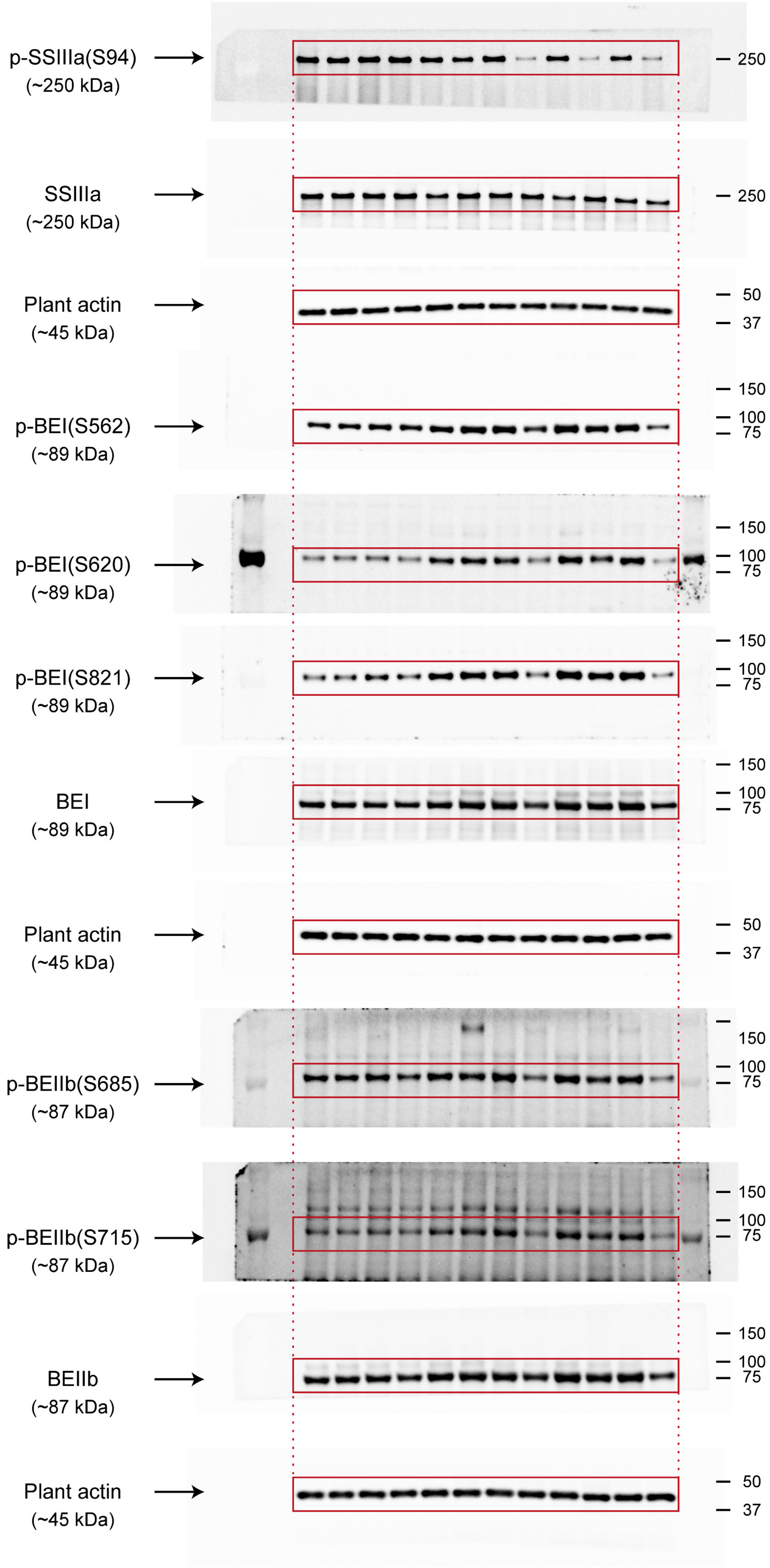
p-SSIIIa(S94)
(~250 kDa)
250
SSIIIa
(~250 kDa)
250
Plant actin
(~45 kDa)
50
37
p-BEI(S562)
(~89 kDa)
150
100
75
p-BEI(S620)
(~89 kDa)
150
100
75
p-BEI(S821)
(~89 kDa)
150
100
75
BEI
(~89 kDa)
150
100
75
Plant actin
(~45 kDa)
50
37
p-BEIIb(S685)
(~87 kDa)
150
100
75
p-BEIIb(S715)
(~87 kDa)
150
100
75
BEIIb
(~87 kDa)
150
100
75
Plant actin
(~45 kDa)
50
37

Figure S20

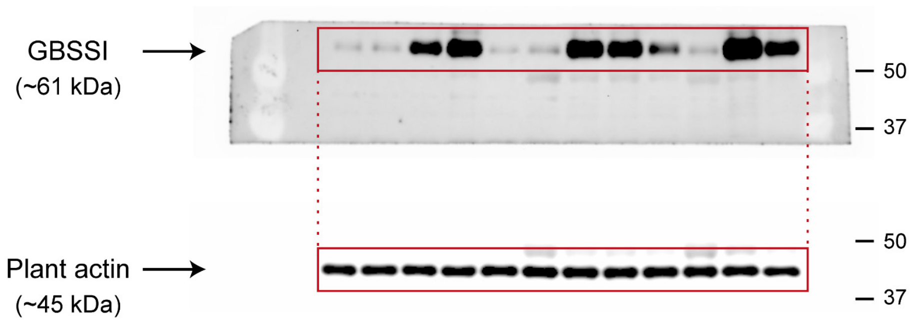

Supplement: Supplementary file 1 [file ijms-22-10546-s001.zip › Supplementary Figures-20210926.pdf]
